# Genetically altered animal models for *ATP1A3*-related disorders

**DOI:** 10.1242/dmm.048938

**Published:** 2021-10-06

**Authors:** Hannah W. Y. Ng, Jennifer A. Ogbeta, Steven J. Clapcote

**Affiliations:** 1 School of Biomedical Sciences, University of Leeds, Leeds LS2 9JT, UK; 2 European Network for Research on Alternating Hemiplegia (ENRAH), 1120 Vienna, Austria

**Keywords:** *ATP1A3*, Na^+^,K^+^-ATPase α_3_, Neurological disorders, Animal models

## Abstract

Within the past 20 years, particularly with the advent of exome sequencing technologies, autosomal dominant and *de novo* mutations in the gene encoding the neurone-specific α_3_ subunit of the Na^+^,K^+^-ATPase (NKA α_3_) pump, *ATP1A3*, have been identified as the cause of a phenotypic continuum of rare neurological disorders. These allelic disorders of *ATP1A3* include (in approximate order of severity/disability and onset in childhood development): polymicrogyria; alternating hemiplegia of childhood; cerebellar ataxia, areflexia, pes cavus, optic atrophy and sensorineural hearing loss syndrome; relapsing encephalopathy with cerebellar ataxia; and rapid-onset dystonia-parkinsonism. Some patients present intermediate, atypical or combined phenotypes. As these disorders are currently difficult to treat, there is an unmet need for more effective therapies. The molecular mechanisms through which mutations in *ATP1A3* result in a broad range of neurological symptoms are poorly understood. However, *in vivo* comparative studies using genetically altered model organisms can provide insight into the biological consequences of the disease-causing mutations in NKA α_3_. Herein, we review the existing mouse, zebrafish, *Drosophila* and *Caenorhabditis elegans* models used to study *ATP1A3*-related disorders, and discuss their potential contribution towards the understanding of disease mechanisms and development of novel therapeutics.

## Abstract (precis)

**Summary:** This Review provides an overview of the animal models used to study the spectrum of *ATP1A3*-related disorders and discusses their contribution towards better understanding of pathological mechanisms and novel therapeutics.

## Introduction

### Na^+^,K^+^-ATPase structure and function

**Fig. 1.**
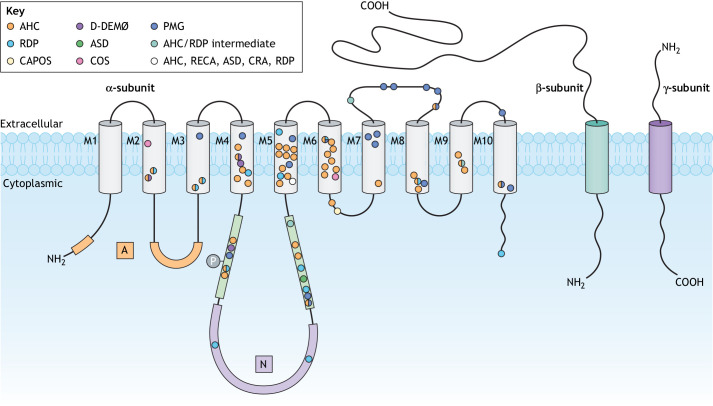
**Location of *ATP1A3*-related disorder mutations.** Schematic of the NKA α3 protein with the cytoplasmic domains (A, N and P), ten transmembrane helices (M1-M10) and extracellular loops. Each circle represents the location of an amino acid residue mutated in *ATP1A3*-related disorders. Different colours represent different disorders. The white circle in M5 indicates R756, mutated in multiple disorders. The encircled ‘P’ indicates the transient phosphorylation of the P-domain aspartate D369. AHC, alternating hemiplegia of childhood; ASD, autism spectrum disorder; CAPOS, cerebellar ataxia, areflexia, pes cavus, optic atrophy and sensorineural hearing loss; COS, childhood-onset schizophrenia; CRA, childhood rapid-onset ataxia; D-DEMØ, dystonia, dysmorphism of the face, encephalopathy with developmental delay, brain MRI abnormalities always including cerebellar hypoplasia, no hemiplegia (Ø); NKA, Na^+^,K^+^-ATPase; PMG, polymicrogyria; RDP, rapid-onset dystonia-parkinsonism; RECA, relapsing encephalopathy with cerebellar ataxia.

The sodium-potassium pump, Na^+^,K^+^-ATPase (NKA), actively transports three Na^+^ ions out of the cell and two K^+^ ions into the cell against their concentration gradients, utilising energy derived from adenosine 5′-triphosphate (ATP) hydrolysis (Kaplan, 2002). The NKA belongs to the P-type ATPase family, so called because the ATPase cycle (known as the ‘Albers-Post’ cycle) is facilitated by the phosphorylation (hence ‘P’) and dephosphorylation of an aspartate residue (Fig. 1) (Albers, 1967; Post et al., 1972). During the cycle, the pump adopts two major conformations, the inward-facing E_1_ state and the outward-facing E_2_ state, which preferentially bind to Na^+^ and K^+^, respectively (Morth et al., 2007; Kanai et al., 2013). The electrochemical ion gradients generated and maintained by the NKA are essential for restoring resting membrane potentials, initiating action potentials and signalling by neurotransmitters (Holm et al., 2016b). Given the importance of NKA function in these cellular processes, it is unsurprising that the pump consumes ∼50% of the energy in the central nervous system (CNS) (Harris et al., 2012).

The NKA consists of two obligatory subunits, a large catalytic α-subunit and a smaller glycosylated auxiliary β-subunit, and occasionally a tissue-specific regulatory γ-subunit from the FXYD protein family that interacts with the αβ heterodimer (Kanai et al., 2013; Kaplan, 2002; Morth et al., 2007). The α-subunit consists of three cytoplasmic domains [nucleotide binding (N), phosphorylation (P) and actuator (A)] and ten transmembrane helices (M1-M10). Mammals express four distinct α-isoforms: α_1_ is expressed ubiquitously; α_2_ is expressed in glial cells (McGrail et al., 1991), the heart (Zahler et al., 1992) and muscles (Hundal et al., 1992); α_3_ is expressed in neurones (McLean et al., 2009; Shamraj and Lingrel, 1994) and the heart (Zahler et al., 1992); and α_4_ is expressed in testis (Woo et al., 2000). NKA α_3_ plays an important role in the rapid restoration of basal intracellular Na^+^ concentrations after high neuronal activity (Azarias et al., 2013). The β-subunit, which is required for NKA maturation, transportation of the αβ complex to the plasma membrane and stabilisation of the E_2_ state, has three isoforms: β_1_, β_2_ and β_3_ (Geering et al., 1996; Geering, 2001; McDonough et al., 1990; Lutsenko and Kaplan, 1993). Although the γ-subunit is not necessary for NKA function (Jones et al., 2005), it modulates the kinetic properties of the enzyme to adapt to the physiological needs of various tissue types (Mishra et al., 2011).

### Human NKA α_3_ mutations

The link between heterozygous missense mutations in the *ATP1A3* gene and the manifestation of rare neurological disorders was first established in 2004 (de Carvalho Aguiar et al., 2004). The human *ATP1A3* gene, comprising 23 exons on chromosome 19q13.2, has alternatively spliced transcript variants encoding three isoforms: variant 1/isoform 1 [3551 bp/1013 amino acids (aa); NM_152296] is the predominant transcript; variant 2/isoform 2 (3466 bp/1024 aa; NM_001256213) has an alternate 5′ exon, resulting in a longer N-terminus; and variant 3/isoform 3 (3590 bp/1026 aa; NM_001256214) has an additional in-frame segment in the 5′ coding region, resulting in a longer protein (Brown et al., 2015). The distribution of, and functional differences between, the *ATP1A3* transcript variants are unknown.

*ATP1A3*-related disorders represent a phenotypic continuum, as different non-synonymous mutations in *ATP1A3* result in a spectrum of clinical syndromes with overlapping symptoms that range broadly in severity, age of onset and progression (Sweney et al., 2015) (Table 1). Mutations causing neonate-onset disorders, such as polymicrogyria (PMG) and alternating hemiplegia of childhood (AHC), are considered to be the most severe (Sweadner et al., 2019). Disorders with onset later in childhood include cerebellar ataxia, areflexia, pes cavus, optic atrophy and sensorineural hearing loss (CAPOS) syndrome and relapsing encephalopathy with cerebellar ataxia (RECA), whereas disorders with onset in adolescence or adulthood include rapid-onset dystonia-parkinsonism (RDP). Some patients present intermediate, atypical or combined phenotypes (Rosewich et al., 2014a; Termsarasab et al., 2015; Sival et al., 2018), a number of which have been given new names, such as D-DEMØ. However, Sival et al. (2018) have cautioned that the assignment of overly exact phenotype-genotype relationships could promote inaccurate interpretation of the overlapping phenotypes within the spectrum of *ATP1A3*-related disorders.

**Table 1.**
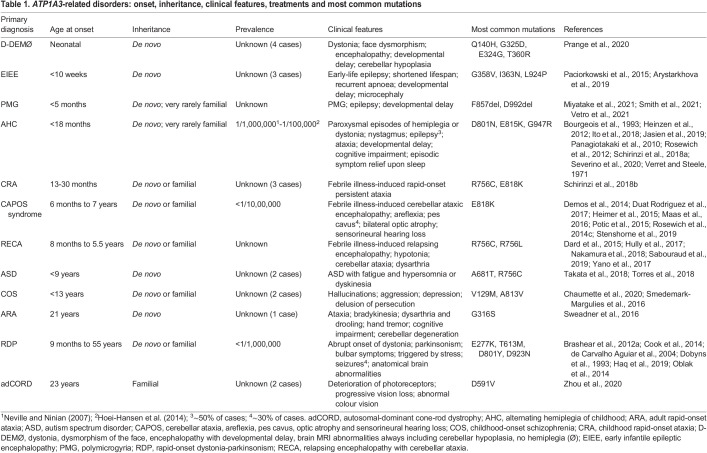
*ATP1A3*-related disorders: onset, inheritance, clinical features, treatments and most common mutations

The positions of mutations within the NKA α_3_ protein (Fig. 1; Tables S1 and S2) have been correlated with phenotypic severity, following the observation that a majority of AHC mutations (∼70%) cluster within and around the ion-binding sites (in M4-M6) relative to mutations associated with milder phenotypes (Sweadner et al., 2019). Pathogenicity studies have shown that *ATP1A3* mutations result in a reduction in catalytic activity and a failure to generate pump current, resulting from a variety of causes, including reduced protein expression, reduced Na^+^ or K^+^ affinity, unusually strong inhibition by K^+^ or an inability of the pump to undergo conformational change (de Carvalho Aguiar et al., 2004; Heinzen et al., 2012; Holm et al., 2016b; Kirshenbaum et al., 2013; Roenn et al., 2019; Simmons et al., 2018; Toustrup-Jensen et al., 2014; Tranebjærg et al., 2018; Weigand et al., 2014).

### *ATP1A3*-related disorders

#### RDP

RDP [DYT12; Online Mendelian Inheritance in Man (OMIM) 128235] was the first disorder identified as being caused by mutations in *ATP1A3* (de Carvalho Aguiar et al., 2004). It is characterised by the sudden onset of dystonia with parkinsonism features and bulbar symptoms, initially affecting the arms or legs in the majority of RDP patients (Haq et al., 2019). The onset of symptoms most commonly occurs in young adulthood and is triggered by stressors including exercise, fever, heat, childbirth and psychological stress (Dobyns et al., 1993; Brashear et al., 2007). Levels of homovanillic acid, a dopamine metabolite, in cerebrospinal fluid (CSF) are diminished in some patients (Brashear et al., 1998), but neuroimaging and pathology do not suggest nigral degeneration (Brashear et al., 1999; Oblak et al., 2014), and dopaminergic medication does not improve symptoms (Brashear et al., 2007). Neuropathology associated with RDP includes atrophy and neuronal loss in parts of the basal ganglia system, brainstem and cerebellum involved in the regulation of movement, motor coordination and motor learning (Oblak et al., 2014).

#### AHC

AHC (OMIM 614820) is an infantile-onset (<18 months) and the most common *ATP1A3*-related disorder, with a reported prevalence of between 1 in 1,000,000 (Neville and Ninian, 2007) and 1 in 100,000 children (Hoei-Hansen et al., 2014). It is characterised by paroxysmal episodes of hemiplegia involving one or both sides of the body, with variable clinical features such as abnormal ocular movements, choreoathetosis and dystonia, and resolution of symptoms with sleep or occlusion of the eyes (Bourgeois et al., 1993; Panagiotakaki et al., 2010; Rosewich et al., 2014a; Verret and Steele, 1971). Like RDP, paroxysmal episodes are often triggered by environmental or physiological stressors (Sweney et al., 2009).

Epileptic seizures occur in ∼50% of AHC patients (Panagiotakaki et al., 2010; Uchitel et al., 2019), and at least 94% of AHC patients exhibit cognitive impairment (Panagiotakaki et al., 2010; Sweney et al., 2009). Among 34 AHC patients evaluated with the Social Responsiveness Scale, 79% showed impaired social skills involving multiple domains and 26% were subsequently diagnosed with comorbid autism spectrum disorder (ASD) (Uchitel et al., 2020). A study of 12 AHC patients revealed reduced total brain and white matter volumes, and a negative correlation between the volume of cerebellar grey matter and severity of ataxia and disability (Severino et al., 2020). Among 87 patients with AHC, 60% exhibited resting electrocardiogram (ECG) abnormalities (Balestrini et al., 2020). Whether AHC is a progressive disorder remains unclear, but a study of 94 patients with AHC detected mild worsening of motor and intellectual disability with age (Uchitel et al., 2021), while abrupt stepwise deterioration (Sasaki et al., 2014) and progressive brain atrophy (Saito et al., 1998; Sasaki et al., 2017) in AHC patients have also been reported. The most common AHC-causing mutations in *ATP1A3* are D801N, E815K and G947R (Table S2), of which E815K has the most severe phenotype (Sasaki et al., 2014; Yang et al., 2014).

#### CAPOS syndrome

CAPOS syndrome (OMIM 601338) patients experience early-onset (<5 years of age) febrile illness-induced relapsing episodes of cerebellar ataxia, usually associated with progressive optic atrophy and sensorineural hearing loss, generalised hypotonia and areflexia. Pes cavus (a foot deformity) affects ∼30% of CAPOS patients (Duat Rodriguez et al., 2017), and cases without pes cavus are sometimes referred to as CAOS (Heimer et al., 2015). CAPOS begins with one to three fever-induced acute episodes of ataxic encephalopathy, from which patients usually recover over days to months, and slow disease progression thereafter (Duat Rodriguez et al., 2017). In CAPOS patients, cochlear outer hair cell activity is preserved, but auditory brainstem responses are grossly abnormal (Tranebjærg et al., 2018). All of the reported cases of CAPOS, to date, are heterozygous for a single recurrent missense mutation, E818K (Demos et al., 2014; Duat Rodriguez et al., 2017; Han et al., 2017; Heimer et al., 2015; Maas et al., 2016; Potic et al., 2015; Rosewich et al., 2014c; Stenshorne et al., 2019). Some CAPOS patients exhibit mild symptoms, resulting in recovery with minimal residual ataxia (Demos et al., 2014), although one subject with a *de novo* E818K mutation exhibited only sensorineural hearing loss that began when she was a teenager (Han et al., 2017).

#### PMG

PMG is the most common developmental malformation of the cerebral cortex, characterised by abnormal folding and laminar organisation. Recently, *de novo* and familial mutations in *ATP1A3* were found in patients affected by a severe form of PMG characterised by epilepsy and a global developmental delay (Miyatake et al., 2021; Smith et al., 2021; Vetro et al., 2021). PMG mutations include D801N, the most common mutation in AHC (Panagiotakaki et al., 2015), and L924P, which is also observed in early infantile epileptic encephalopathy (EIEE) (Arystarkhova et al., 2019). A human *ATP1A3* complementary DNA (cDNA) construct harbouring the mutation D992del, observed in two patients with severe PMG, was found to impair radial neuronal migration in the cerebral cortex of C57BL/6 mice at embryonic day (E)18.5, after being introduced into the ventricles on E14.5 by *in utero* electroporation (Miyatake et al., 2021), suggesting that *ATP1A3*^D992del^ causes defects in cortical architecture.

#### RECA

RECA is characterised by recurrent neurological decompensation episodes triggered by febrile illness, leading to encephalopathy with acute cerebellar ataxia and occasionally chorea, dystonia, dysarthria, mutism and dysphagia (Dard et al., 2015; Hully et al., 2017; Nakamura et al., 2018; Sabouraud et al., 2019). To date, all RECA cases are associated with substitutions of arginine 756 (R756H, R756C) (Dard et al., 2015; Hully et al., 2017; Jaffer et al., 2017; Nakamura et al., 2018; Nicita et al., 2016; Sabouraud et al., 2019). A very similar *ATP1A3*-related disorder, febrile-induced paroxysmal weakness and encephalopathy (FIPWE), is also caused by mutations at R756 (Yano et al., 2017). Owing to the genetic and phenotypic overlaps between FIPWE and RECA (Sabouraud et al., 2019), in this Review, we ascribe the term RECA to all FIPWE cases.

#### Autosomal-dominant cone-rod dystrophy

Autosomal-dominant cone-rod dystrophy (adCORD) is an inherited retinal degenerative disorder characterised by progressive deterioration of cone and rod photoreceptors. Affected individuals typically present with progressive loss of vision, reduced visual acuity and problems with colour vision from the age of 12 (Thiadens et al., 2012). The *ATP1A3* mutation D591V has been identified in a single family affected by a form of adCORD (Zhou et al., 2020).

#### Other *ATP1A3*-related clinical phenotypes

Mutations in *ATP1A3* have also been identified in individuals presenting with other rare clinical phenotypes. D-DEMØ, observed in four patients, consists of dystonia, dysmorphism of the face, encephalopathy with developmental delay and magnetic resonance imaging (MRI) brain abnormalities (cerebellar hypoplasia) (Prange et al., 2020). EIEE, observed in three patients, is characterised by seizures beginning within the first few days after birth, resulting in the death of two patients in infancy (Paciorkowski et al., 2015; Arystarkhova et al., 2019). Childhood rapid-onset ataxia (CRA), observed in three patients, is characterised by the presentation of ataxic features following a febrile episode during infancy (<36 months); two patients have the R756C mutation associated with RECA and the third has the E818K mutation associated with CAPOS syndrome (Schirinzi et al., 2018b). Adult rapid-onset ataxia (ARA) has been observed in one patient, with a *de novo* G316S mutation, in whom difficulties with balance and gait emerged at 21 years (Sweadner et al., 2016). *ATP1A3* mutations have also been identified in individuals with forms of ASD (Takata et al., 2018; Torres et al., 2018; Chaumette et al., 2020) and childhood-onset schizophrenia (COS) (Smedemark-Margulies et al., 2016; Chaumette et al., 2020).

## Animal models of *ATP1A3*-related neurological disorders

### Mouse

Mouse models are valuable tools to study *ATP1A3*-related disorders, owing to their high genomic similarity to humans. The mouse orthologue, *Atp1a3*, comprising 23 exons on chromosome 7, has 99.6% protein and 90.8% DNA identity relative to human *ATP1A3* (NCBI HomoloGene 113729) (Table 2). *Atp1a3* has alternatively spliced transcript variants encoding two isoforms: variant 1/isoform 1 (3615 bp/1026 aa; NM_001374627) and variant 2/isoform 2 (3609 bp/1013 aa; NM_001290469). Several mouse models to study the *in vivo* consequences of NKA α_3_ mutations have been published to date (Table 3). The seven behavioural tests most commonly used to assess mouse models of *ATP1A3*-related disorders are briefly described in Box 1.

**Box 1. Behavioural tests in mouse models of *ATP1A3*-related disorders**

**Learning and memory**

**Table 2.**
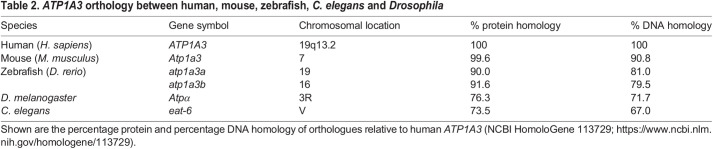
*ATP1A3* orthology between human, mouse, zebrafish, *C. elegans* and *Drosophila*

**Table 3.**
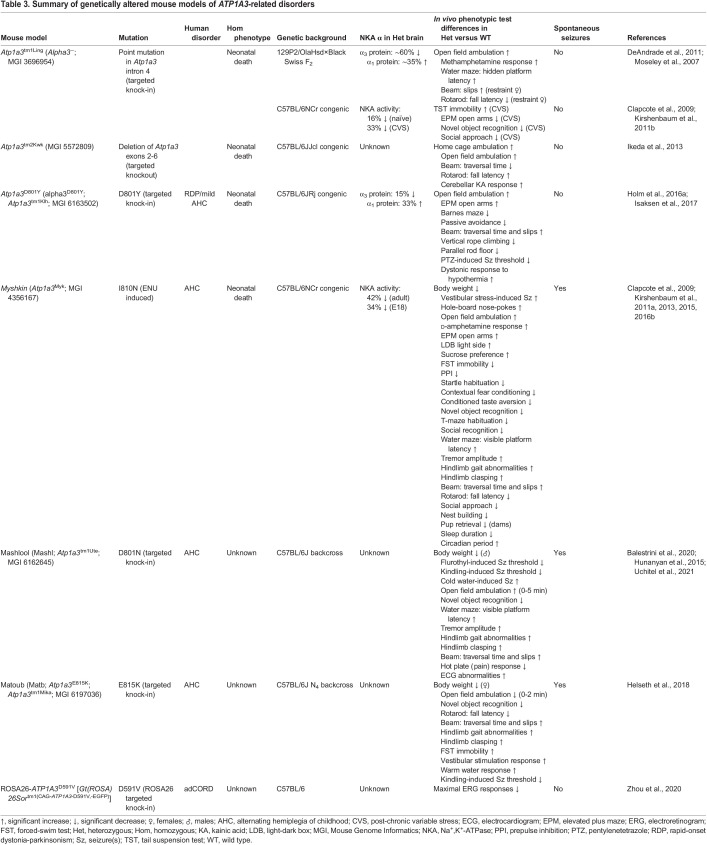
Summary of genetically altered mouse models of *ATP1A3*-related disorders

**Morris water maze:** the hidden platform (spatial) version is a test of spatial learning and memory, in which the mouse is placed in a large circular tub of water and required to use extramaze visual cues to navigate to a submerged escape platform. The visible platform version is sometimes used as a non-spatial control task, in which the escape platform is indicated by a marker above the water surface.

**Novel object recognition:** the mouse is presented with two identical objects during the training session, and then one of the two objects is replaced by a novel object during a test session. The amount of time taken exploring the novel object provides a measure of recognition memory.

**Motor function and locomotor activity**

**Beam-walking:** the goal of the balance beam test is for the mouse to stay upright and walk across an elevated narrow beam to a safe platform. The number of errors (foot slips) and latency to cross the beam are recorded as measures of motor skills and balance.

**Gait analysis:** the fore and hind paws of the mouse are coated with two colours of non-toxic paint, and then the mouse is allowed to walk along a narrow, paper-covered runway. The width and length of the fore stride and hind stride are measured from the resulting pawprint patterns.

**Hindlimb clasping:** the mouse is suspended by the tail for 30 s and observed for hindlimb clasping (retraction of either or both hindlimbs into the body and toward the midline), a behaviour indicative of general neurological dysfunction.

**Open field:** the open field consists of a square wall-enclosed arena into which the mouse is placed and allowed to move about freely for 10-60 min while being recorded by an overhead camera. The test is used to determine various parameters of locomotor activity (e.g. ambulation) and exploration habits (e.g. time at periphery versus centre).

**Rotarod:** the mouse is placed on a horizontally oriented rod that begins rotating at a constant or accelerating speed. The latency to fall onto a platform below, and the speed of rod rotation upon falling, are recorded as measures of balance and coordination.

#### *Atp1a3* ^tm1/Ling^

The *Atp1a3*^tm1/Ling^ model [Alpha3^−^; Mouse Genome Informatics (MGI) 3696954] was the first-described mouse line harbouring a mutation of the *Atp1a3* gene (Moseley et al., 2007). The line was generated via homologous recombination in 129P2/OlaHsd-derived (mEMS32) embryonic stem (ES) cells that introduced a point mutation into *Atp1a3* intron 4 adjacent to the exon-intron splice site, resulting in aberrant splicing, adding 126 bp to the transcript. Homozygous *Atp1a3*^tm1/Ling^ mice die neonatally and show a very low level of NKA α_3_ mRNA in the foetal brain compared with wild-type (WT) mice, such that *Atp1a3*^tm1/Ling^ is not a complete null. Heterozygous mice (*Atp1a3*^tm1/Ling/+^) have a ∼60% reduction in NKA α_3_ protein levels in the hippocampus (Moseley et al., 2007) and a 16% reduction in brain NKA activity (contributed by α_1_+α_2_+α_3_ subunits) (Clapcote et al., 2009).

*Atp1a3*^tm1/Ling/+^ mice with a 129P2/OlaHsd×Black Swiss F_2_ genetic background displayed increased locomotor activity in the open field and after administration of methamphetamine (a drug that elevates levels of extracellular monoamine neurotransmitters in the brain), as well as spatial learning and memory deficits in the Morris water maze (Moseley et al., 2007). Restraint-stress induced motor deficits in female *Atp1a3*^tm1/Ling/+^ mice in the beam-walking and rotarod tests, but non-stressed females were unaffected (DeAndrade et al., 2011). Grip strength in stressed *Atp1a3*^tm1/Ling/+^ mice was intact, suggesting that motor deficits were not of a muscular origin. Male *Atp1a3*^tm1/Ling/+^ mice did not display restraint stress-induced motor deficits (DeAndrade et al., 2011), indicating a sex-dependent effect.

*Atp1a3*^tm1/Ling/+^ mice with a C57BL/6NCrl congenic background were exposed to chronic variable stress (CVS) by subjecting them to unpredictable mild stressors daily for 6 weeks (Kirshenbaum et al., 2011b). CVS-treated *Atp1a3*^tm1/Ling/+^ mice displayed 33% reduced brain NKA activity (versus 16% reduction in naïve mice), which was associated with increased anxiety in the elevated plus maze, impaired long-term memory in the novel object recognition test and reduced preference for social novelty compared with CVS-treated WT mice. This demonstrates that the interaction of environmental stress and NKA α_3_ mutation can have a negative impact on brain NKA activity, with consequent effects on behaviour. CVS-treated *Atp1a3*^tm1/Ling/+^ and WT mice both exhibited despair-like behaviour in the tail suspension test, and anhedonia in the sucrose preference test. No sex differences were reported (Kirshenbaum et al., 2011b).

#### *Atp1a3* ^tm2Kwk^

The *Atp1a3*^tm2Kwk^ mouse model (MGI 5572809) was generated via homologous recombination in C57BL/6NCrl×CBA/J F_1_-derived (TT2) ES cells by replacing *Atp1a3* exons 2-6 with a loxP-flanked *Atp1a3*-enhanced green fluorescent protein (EGFP) fusion protein, which was subsequently deleted by Cre-mediated recombination (Ikeda et al., 2013). Homozygous *Atp1a3*^tm2Kwk^ mice die neonatally, owing to a complete lack of breathing movements. Heterozygous (*Atp1a3*^tm2Kwk/+^) mice with a C57BL/6JJcl congenic background did not exhibit obvious motor deficits, spontaneously or after exposure to stressors (tail suspension, forced swimming or restraint stress). *Atp1a3*^tm2Kwk/+^ mice did, however, display increased locomotor activity in the open field, and performed significantly better than WT mice in the beam-walking and rotarod tests. Injection of kainic acid (KA; a convulsant) into the cerebellum, a brain region important in motor control, induced dystonia in both *Atp1a3*^tm2Kwk/+^ and WT mice, but heterozygotes had a longer dystonic response and recovery time (Ikeda et al., 2013). Ikeda et al. (2013) also observed altered neurotransmission across inhibitory synapses in *Atp1a3*^tm2Kwk/+^ mice, suggesting that perturbed inhibitory circuits may have a potential role in the manifestation of dystonia.

In an evaluation of social behaviours, *Atp1a3*^tm2Kwk/+^ mice showed a lower degree of social interaction, a lower frequency of barbering (removal of whiskers and facial hair) and a lower hierarchical rank (a reflection of social dominance) within a mixed-genotype cage relative to WT mice (Sugimoto et al., 2018).

Levels of ascorbic acid (vitamin C; a vital antioxidant) were found to be decreased in the basal ganglia and cerebellum of *Atp1a3*^tm2Kwk/+^ mice, and were decreased further in *Atp1a3*^tm2Kwk/+^ dams within 12 h of giving birth, compared with WT controls (Ikeda et al., 2021). Perinatal *Atp1a3*^tm2Kwk/+^ dams also displayed transient dystonic episodes (Ikeda et al., 2021), paralleling the worsening of symptoms observed in CAPOS syndrome during pregnancy and in the immediate postpartum period, when maternal body temperature is raised (Chang et al., 2018). Thus, although the *Atp1a3*^tm1/Ling^ and *Atp1a3*^tm2Kwk^ models do not replicate specific mutations found in *ATP1A3*-related disorders, they have both demonstrated utility for investigating trigger-induced symptoms.

#### Myshkin

The *Myshkin* mouse model (*Atp1a3*^Myk^; MGI 4356167) harbouring mutation I810N in *Atp1a3* exon 18 was originally identified as a phenodeviant C57BL/6J×129S1/SvImJ F_1_ mouse with an altered phenotype in an N-nitroso-N-ethylurea (ENU; a chemical mutagen) mutagenesis screen (Clapcote et al., 2009). The I810N mutation has been observed in three AHC patients, including one with comorbid ASD, all of whom presented with typical AHC symptoms and epilepsy (Panagiotakaki et al., 2015; Yang et al., 2014, 2015)*.* Homozygous *Myshkin* mice die neonatally and show a 56% reduction in brain NKA activity at E18.5 compared with WT littermates. Adult heterozygous *Myshkin* (*Myk*/+) mice backcrossed 12 generations (N_12_) to the C57BL/6NCr strain have a 42% reduction in NKA enzyme activity in the adult brain and a 16-18% reduction in body weight compared with WT mice (Clapcote et al., 2009). *Myk*/+ mice exhibit an elevated metabolic rate, a visible whole-body tremor and a broad-based gait (Kirshenbaum et al., 2013; Timothy et al., 2018). 2-Deoxy-d-glucose imaging identified a deficit in frontal cortex functioning (hypofrontality) in the *Myk*/+ mouse brain, directly mirroring that reported in AHC (Sasaki et al., 2009; Sweney et al., 2009), along with reduced thalamocortical functional connectivity (Kirshenbaum et al., 2013).

*Myk*/+ mice exhibit spontaneous and vestibular stress-induced seizures, medial temporal sclerosis, sleep abnormalities, and a variety of motor, cognitive, social and other behavioural deficits (Table 3) (Clapcote et al., 2009; Kirshenbaum et al., 2011a, 2013, 2015, 2016b) consistent with the multiple comorbidities in AHC (Jasien et al., 2019; Kansagra et al., 2019; Neville and Ninan, 2007). In hippocampal slices from *Myk*/+ mice, the CA3-CA1 hippocampal pathway showed normal levels of synaptic transmission, plasticity and excitability under basal conditions, but became hyperexcitable after high-frequency synaptic activity (Clapcote et al., 2009), such as that precipitated by stress, consistent with the role of NKA α_3_ in the rapid extrusion of Na^+^ after high neuronal activity (Azarias et al., 2013).

The seizures of *Myk*/+ mice are occasionally fatal, but their severity was reduced by valproate (an anticonvulsant), and they were prevented by inheritance of a WT *Atp1a3* cDNA transgene, conferring a 16% increase in brain NKA activity (Clapcote et al., 2009). The WT *Atp1a3* cDNA also rescued *Myshkin* body weight, motor dysfunction, contextual fear conditioning and mania-like behaviours (greater risk-taking and impulsivity) (Kirshenbaum et al., 2011a, 2016a). Attenuation of mania-like behaviours was also observed following treatment with lithium and valproate (mood-stabilisers), SL327 (an ERK inhibitor) and rostafuroxin (an antagonist of the NKA blocker ouabain) (Kirshenbaum et al., 2011a).

Compared with N_12_ C57BL/6NCr animals, *Myk*/+ mice backcrossed 20 generations to C57BL/6NCr (N_20_) showed a significantly lower reduction in adult brain NKA activity (36% versus 42%) and did not exhibit seizure activity in electrocorticography recordings when subjected to vestibular stress (Kirshenbaum et al., 2011a). This demonstration of phenotypic modification by the genetic background is supported by the observation that two crosses (N_2_) to the seizure-susceptible FVB/NCr strain (Goelz et al., 1998) resulted in significant levels of focal reactive astrogliosis and microglial activation in the hippocampus that were not observed in N_12_ C57BL/6NCr *Myk*/+ mice (Clapcote et al., 2009).

#### *Atp1a3* ^D801Y^

The *Atp1a3*^D801Y^ mouse model (alpha3^D801Y^; *Atp1a3*^tm1Klh^; MGI 6163502) was generated via homologous recombination in 129S1/SvImJ-derived (CJ7) ES cells that replaced *Atp1a3* exon 17 with a modified exon 17 harbouring the D801Y mutation (Holm et al., 2016a). A variety of mutations affecting the aspartic acid at position 801 (D801) can cause RDP, AHC or PMG. D801E and D801V are AHC-causing mutations (Panagiotakaki et al., 2015), whereas D801N can cause AHC and PMG (Panagiotakaki et al., 2015; Vetro et al., 2021), and D801Y can cause RDP and AHC (de Carvalho Aguiar et al., 2004; Viollet et al., 2015). Homozygous *Atp1a3*^D801Y^ mice die neonatally. Heterozygous (*Atp1a3*^D801Y/+^) mice with a C57BL/6JRj congenic background have a 15% reduction in total NKA α3 protein levels in the brain (Holm et al., 2016a).

*Atp1a3*^D801Y/+^ mice display increased locomotor activity in the open field, a lower threshold for seizures induced by pentylenetetrazole (PTZ; a convulsant) and learning and memory impairments in the Barnes maze and passive avoidance test, of which the latter was rescued by administration of clonazepam (a tranquiliser that enhances GABAergic inhibition) (Holm et al., 2016a). Although gait, posture and grip strength are not significantly different in *Atp1a3*^D801Y/+^ mice, beam-walking, vertical rope climbing and parallel rod floor tests revealed moderate motor deficits (Isaksen et al., 2017). *Atp1a3*^D801Y/+^ mice also display hypothermia-induced dystonia after cold water (5-10°C) swimming for 4 min or cold environment (−20°C) exposure, consistent with dystonic episodes triggered by temperature changes in RDP patients (de Carvalho Aguiar et al., 2004; Isaksen et al., 2017). Other stressors, including forced swimming in warm (35°C) water, high temperature (43°C) for 15 min, restraint stress and foot shocks, do not induce dystonia in *Atp1a3*^D801Y/+^ mice (Isaksen et al., 2017).

#### Mashlool

The Mashlool mouse model (Mashl; *Atp1a3*^tm1Ute^; MGI 6162645) was generated via homologous recombination in 129S1/SvImJ×129X1/SvJ F_1_-derived (R1) ES cells that replaced *Atp1a3* exon 17 with a modified exon 17 harbouring the D801N mutation (Hunanyan et al., 2015). D801N is the mutation most commonly observed in humans with AHC (Table S2). *In vitro* studies involving heterologous overexpression of *ATP1A3* have shown that D801N does not affect protein levels but reduces ATPase activity by 54-80% (Heinzen et al., 2012). Heterozygous Mashlool (Mashl/+) mice with a C57BL/6J backcross background exhibit reduced body weight in males (by ∼11%) but not females (Hunanyan et al., 2015).

In the open field, Mashl/+ mice display increased locomotor activity and time at the centre of the arena during the first 5 min but not thereafter (Hunanyan et al., 2015). Mashl/+ mice also display impaired long-term memory in the novel object recognition test and motor dysfunction in the gait, beam-walking and hindlimb clasping tests. During these experiments, 40% of Mashl/+ mice exhibited transient (∼90-s duration) hemiplegia or hemiparesis, replicating a core symptom of AHC. Mashl/+ mice also exhibit a lower threshold for seizures induced by amygdala kindling (focal electrical stimulation) and flurothyl (a convulsant). Spontaneous recurrent and occasionally fatal seizures were also observed in both kindled and non-kindled mutant mice (Hunanyan et al., 2015).

Resting ECGs in Mashl/+ mice revealed an increased heart rate and intracardiac conduction delay compared with WT mice (Balestrini et al., 2020). After injection of KA into the amygdala, Mashl/+ mice show ECG abnormalities in response to seizure activity (Balestrini et al., 2020). Compared with young (5-week-old) Mashl/+ mice, adult (17-week-old) Mashl/+ mice exhibit an inferior beam-walking performance and an increased severity of seizures and mortality induced by cold water (5-10°C) swimming for 4 min (Uchitel et al., 2021), suggestive of disease progression.

In hippocampal slices from Mashl/+ mice, the CA3-CA1 hippocampal pathway shows hyperexcitable responses to repetitive high-frequency stimulation (Hunanyan et al., 2015), similar to those observed in *Myk*/+ mice (Clapcote et al., 2009). This increased hippocampal excitability is due to decreased GABA_A_ receptor-mediated inhibition, consistent with a reduced number of parvalbumin-expressing inhibitory interneurones in the hippocampal CA1 in Mashl/+ mice (Hunanyan et al., 2018). The application of extracellular potassium induces greater spreading depression (a transient wave of depolarisation) in Mashl/+ slices than in WT slices (Hunanyan et al., 2015), consistent with previous work demonstrating prolonged spreading depression in response to ouabain (Haglund and Schwartzkroin, 1990).

An adeno-associated virus serotype 9 (AAV9) vector expressing human *ATP1A3* cDNA under the control of a human *SYN1* (neurone-specific) promoter, which had been shown to increase brain NKA activity in WT mice, was injected into the CSF, via the cerebral ventricles and cisterna magna, of Mashl/+ mice on postnatal day (P)10 (Hunanyan et al., 2021). This exogenous delivery of WT *ATP1A3* to Mashl/+ mice resulted in a reduced occurrence of hemiplegic but not dystonic episodes induced by cold water swimming at P40, improvement in beam-walking performance at P40 and P70 (10 weeks) and prolonged survival compared with untreated Mashl/+ mice up to 10 weeks of age. Although other behavioural tests did not reveal differences in treated Mashl/+ mice (Hunanyan et al., 2021), these results demonstrate that gene therapy can ameliorate some of the manifestations of AHC in the Mashlool model.

#### Matoub

The Matoub mouse model (Matb; *Atp1a3*^E815K^; *Atp1a3*^tm1Mika^; MGI 6197036) was generated via homologous recombination in 129S1/SvImJ×129X1/SvJ F_1_-derived (R1) ES cells by replacing *Atp1a3* exon 18 with a modified exon 18 harbouring the E815K mutation (Helseth et al., 2018). E815K is the second most common mutation in patients with AHC (Table S2) and has a more severe phenotype than that of D801N and G947R heterozygotes. E815K heterozygous AHC patients have an earlier onset of disease and seizures, more frequent status epilepticus, plegic attacks and autonomic dysfunction, and more severe cognitive and motor deficits (Ishii et al., 2013; Panagiotakaki et al., 2015; Sasaki et al., 2014; Viollet et al., 2015; Yang et al., 2014).

Heterozygous (Matb/+) mice with a C57BL/6J N_4_ backcross background manifest episodes of hemiplegia or seizures around the time of weaning at 3-4 weeks of age. Female, but not male, Matb/+ mice exhibit reduced body weight (by ∼29%). Within a cohort of mice monitored up to 12 months, Matb/+ individuals showed increased mortality, with ∼33% dying during a witnessed seizure and none surviving beyond 9 months of age. Matb/+ mice also displayed impaired long-term memory in the novel object recognition test and motor dysfunction in the gait, rotarod, beam-walking and hindlimb clasping tests (Helseth et al., 2018).

After being subjected to 1 min of forced swimming in 40°C water, warmer than the body temperature associated with a febrile state in humans (37.5-39°C) (Del Bene, 1990), 75% of Matb/+ mice displayed an episode of hemiplegia. The duration of the hemiplegia was shortened by treatment with flunarizine (Helseth et al., 2018), a calcium channel blocker that reduces the severity of hemiplegic attacks in some AHC patients (Sasaki et al., 2001; Ito et al., 2018). However, retesting of Matb/+ mice 35 and 98 days after the last injection indicated no long-term beneficial effect of flunarizine treatment (Helseth et al., 2018).

#### ROSA26-*ATP1A3*^D591V^

The ROSA26-*ATP1A3*^D591V^ mouse model [*Gt(ROSA)26Sor*^tm1(CAG-*ATP1A3*-D591V,-EGFP)^], carrying the mutation D591V in *ATP1A3* identified in adCORD, was generated via homologous recombination in C57BL/6-derived ES cells (Zhou et al., 2020). However, the endogenous mouse *Atp1a3* gene was left intact. Instead, a human *ATP1A3* cDNA harbouring D591V, under the control of the CMV early enhancer/chicken β-actin (CAG) promoter, was introduced into the mouse *Gt(ROSA)26Sor* locus (Zhou et al., 2020), which is a widely used site for the integration of transgenes. The CAG promotor drives strong and ubiquitous gene expression, in contrast to the *Atp1a3* promoter that drives selective expression in neurones.

Heterozygous ROSA26*-ATP1A3*^D591V/+^ mice with a C57BL/6 genetic background had unaltered electroretinogram (ERG) responses and retinal cell morphology at 3 months of age (young adult), but maximal ERG responses were decreased at 12 months, indicative of cone and rod dysfunction (Zhou et al., 2020). ROSA26*-ATP1A3*^D591V/+^ mice thus had a comparatively later onset and less severe retinal dysfunction than that exhibited by D591V heterozygous adCORD patients (Zhou et al., 2020). This could be because, unlike D591V/+ heterozygous adCORD patients, both copies of the NKA α3 gene are intact in ROSA26*-ATP1A3*^D591V/+^ mice.

### Zebrafish (*Danio rerio*)

Zebrafish have two *ATP1A3* orthologues, *atp1a3a* and *atp1a3b* (Rajarao et al., 2001), which have a 94.5% amino acid sequence identity with each other. Moreover, the proteins are 90.0% (*atp1a3a*) and 91.6% (*atp1a3b*), and the DNA is 81.0% (*atp1a3a*) and 79.5% (*atp1a3b*), identical relative to human *ATP1A3* (NCBI HomoloGene 113729) (Table 2). Similar to mammalian NKA α_3_, *atp1a3a* and *atp1a3b* are predominantly expressed in the brain (Doğanlı et al., 2013). Zebrafish embryos serve as a promising bridge model between *in vitro* and *in vivo* research, with the potential to replace full-grown animals in experimentation. Expression of the *atp1a3a* transcript is highest in zebrafish embryos at 60 h post-fertilisation (hfp) and is found in various CNS structures, including the epiphysis, tegmentum, tectum, cerebellum, cranial ganglia, hindbrain and spinal cord (Doğanlı et al., 2013). By contrast, expression of the *atp1a3b* transcript is observed in both 60 hpf zebrafish embryos and adults. Within embryos, *atp1a3b* is localised to similar CNS regions as *atp1a3a*, excluding the tectum and posterior spinal cord (Doğanlı et al., 2013).

Splice-blocking morpholino antisense oligonucleotides (SP-MOs) and translation-blocking morpholino antisense oligonucleotides (MOs), which cause mRNA knockdown (KD) by nonsense-mediated mRNA decay or inhibition of protein synthesis, respectively, have facilitated the study of NKA α_3_ in zebrafish embryos (Doğanlı et al., 2013). The majority of embryos injected with *atp1a3a*-SP-MO or *atp1a3b*-SP-MO exhibit a reduced level of *atp1a3a* (∼62%) or *atp1a3b* (∼66%) transcript and show severe hydrocephalus as a consequence of CSF build-up within the brain ventricle (Doğanlı et al., 2013). Meanwhile, the majority of *atp1a3a*-MO- and *atp1a3b*-MO-injected embryos showed a mild hydrocephalus phenotype. This phenotype was rescued in a significant proportion of KD embryos by co-injection of WT *atp1a3a* mRNA (but not WT *atp1a3b* mRNA) in *atp1a3a* KD embryos, and WT *atp1a3b* mRNA (but not WT *atp1a3a* mRNA) in *atp1a3b* KD embryos (Doğanlı et al., 2013). Although hydrocephalus is not typically observed in patients with *ATP1A3*-related disorders, this zebrafish finding raises the possibility that NKA α_3_ is involved in regulating brain volume (Doğanlı et al., 2013). Zebrafish embryos may thus provide insight into the smaller total brain volume observed in both AHC and EIEE patients (Paciorkowski et al., 2015; Severino et al., 2020). In response to tactile stimulation of the embryo trunk, *atp1a3a* KD embryos and *atp1a3b* KD embryos (60 hpf) both display brief distance recoils and occasional convulsions, which are delayed in *atp1a3b* KD embryos, in contrast to the burst swimming exhibited by control embryos (Doğanlı et al., 2013). These observations underline the importance of NKA α_3_ in motor control.

### Fruit fly (*Drosophila melanogaster*)

The *D. melanogaster ATPalpha* (*Atpα*) gene (FlyBase ID FBgn0002921) is responsible for encoding the Na^+^ pump α subunit, which is orthologous to all four α-subunits expressed in mammals, such that *Atpα* is not a specific one-to-one orthologue of *ATP1A3*. The catalytic α-subunit in the fruit fly is expressed ubiquitously, unlike the selective expression of *ATP1A3* in neurones, and has a protein identity of 76.3% and a DNA identity of 71.7% relative to human *ATP1A3* (NCBI HomoloGene 113729) (Table 2). All the residues known to be mutated in *ATP1A3*-related disorders, apart from Q895, are conserved in the *Drosophila* α subunit (Tables S1 and S2). Mutations in *Atpα*, affecting highly conserved amino acid residues, have been generated through ethylmethanesulfonate (EMS) mutagenesis. The *Drosophila* mutant *Atpα^CJ10^* (*CJ10*) carries an amino acid change (G744S) affecting glycine 744 in *Atpα*, which is conserved as glycine 755 in *ATP1A3* and mutated to G755A, G755C, G755S and G755V in patients with AHC (Sasaki et al., 2014; Viollet et al., 2015). The *Drosophila* mutant *Atpα^DTS2^* (*DTS2*) carries an amino acid change (D981N) affecting aspartic acid 981 in *Atpα* (Ashmore et al., 2009), which is conserved as aspartic acid 992 in *ATP1A3* and deleted (D992del) and duplicated (D992dup) in PMG (Table S1) and mutated to D992Y in AHC (Table S2).

*Atpα* mutations at both G744 and D981 are homozygous lethal but lead to AHC-relevant phenotypes in heterozygous adults (Palladino et al., 2003; Ashmore et al., 2009). Heterozygous *CJ10* (*CJ10*/+; G744S) flies exhibit deficits in total locomotor activity, total time active and locomotor activity following startle stimulation compared with age-matched WT controls (Ashmore et al., 2009). Following mechanical disturbance via vortexing, *CJ10*/+ flies show an age-dependent mechanical stress-induced paralysis, in which the fly lies on its back with little effective movement of the legs and wings, with a longer duration in older flies (30 days post-eclosion) than younger flies (3 and 10 days post-eclosion) (Ashmore et al., 2009). Heterozygous *DTS2* (*DTS2*/+; D981N) flies display an age-dependent decrement in locomotor activity, becoming sedentary, with a premature loss of walking activity and flight ability (Palladino et al., 2003). *DTS2*/+ flies also exhibit mechanical stress-induced paralysis, but only when maintained at an elevated temperature (28°C), rather than room temperature (20-22°C) (Palladino et al., 2003). Upon acute exposure to an even higher ambient temperature (37-38°C), *CJ10*/+ and *DTS2*/+ flies exhibit a reversible heat-induced paralysis (Palladino et al., 2003; Ashmore et al., 2009), paralleling the heat sensitivity of symptoms in *ATP1A3*-related disorders.

Consistent with the severity of the age-dependent stress-sensitive locomotor impairments observed in *CJ10*/+ and *DTS2*/+ flies, comparisons of the time required to reach 50% survivorship on their respective lifespan curves (median lifespan) relative to WT flies reveal that both *Atpα* mutants are comparatively short lived (Palladino et al., 2003; Ashmore et al., 2009). This shortened lifespan and the progressive loss of motor activity exhibited by *CJ10*/+ and *DTS2*/+ flies are consistent with the phenotypes exhibited by other *Drosophila* mutants known to be associated with neurodegeneration (Min and Benzer, 1997). Histological analysis revealed no evidence of neurodegeneration in young *Atpα* mutants, but *CJ10*/+ and *DTS2*/+ flies aged to median lifespan exhibited loss of brain tissue, indicated by the accumulation of vacuolar and spongiform-like neuropathology in the central brain and optic lobe, in contrast to the minimal neuropathology seen in aged WT controls (Palladino et al., 2003; Ashmore et al., 2009). Thus, neurodegeneration in these *Atpα* mutants appears to be age dependent, paralleling the progressive brain atrophy observed in AHC patients (Saito et al., 1998; Sasaki et al., 2017).

In a genome-wide screen for novel modifier loci, using 358 deficiency strains, each having a unique deletion of a segment of the genome, Talsma et al. (2014) identified 29 loci that modified the mechanical stress-induced paralysis of *CJ10*/+ flies at 15 days post-eclosion. Within these loci, classical mutants and transgenic RNA interference were used to identify 33 genes that, when disrupted, suppressed (32 genes) or enhanced (one gene) the paralysis phenotype. Suppressors include *Ppk11*, *Ppk21* and *Ppk24*, which encode ligand-gated Na^+^ channels (Talsma et al., 2014). Those suppressor genes that encode proteins expressed in the adult fly offer proteins/pathways that could be viable targets for the development of new drugs for AHC and other *ATP1A3-*related disorders.

A limitation of *Drosophila Atpα* mutants for modelling *ATP1A3*-related disorders is that their NKA α-subunit dysfunction is not restricted to neurones. An alternative, transgenic approach would employ the Gal4-UAS system (Brand and Perrimon, 1993) to generate flies with neurone-specific expression of a human mutant *ATP1A3* cDNA construct. Using the Gal4-UAS system, Paul et al. (2007) demonstrated that ubiquitous expression of rat *Atp1a1* cDNA could prevent the homozygous embryonic lethality of the *Drosophila* mutant *Atpα^DTS2R^*^3^, with amino acid changes E39A and L346F (Palladino et al., 2003), indicating that rat NKA α_1_ can form a functional αβ complex in the fly context. Similarly, Gal4-UAS transgenic flies with expression of mutant (D369N) *Atpα* restricted to NKA β_2_ subunit (*nrv2*)-positive cells showed increased sensitivity to 1 mM ouabain compared with transgenic flies expressing WT *Atpα* and WT flies (Sun et al., 2001).

### Nematode (*Caenorhabditis elegans*)

The sodium/potassium-transporting ATPase subunit alpha gene, *eat-6*, in *C. elegans* roundworms is the one-to-one orthologue of the *ATP1A3* gene in *Homo sapiens*, with a protein identity of 73.5% and a DNA identity of 67.0% (NCBI HomoloGene 113729) (Table 2). Moreover, almost all the residues known to be mutated in *ATP1A3*-related disorders are conserved in the *C. elegans* EAT-6 protein (Tables S1 and S2). To model the *ATP1A3* G316S mutation found in ARA, the glycine at position 304 of EAT-6, corresponding to NKA α_3_ G316, was mutated to serine in two independent *eat-6* alleles, *eat-6*^rt251^ and *eat-6*^rt252^, using CRISPR/Cas9-mediated gene editing (Sorkaç et al., 2016). Worms homozygous for the *eat-6*^rt251^ and *eat-6*^rt252^ alleles were viable (Sorkaç et al., 2016), indicating that the G304S mutation does not cause a complete loss of function. This is consistent with *in vitro* studies by Sweadner et al. (2016), suggesting that G316S is a hypomorphic allele of NKA α_3_ resulting in decreased function.

As young adults (3 days old), heterozygous *eat-6*^rt252^ (*eat-6*^rt252/+^), but not heterozygous *eat-6*^rt251^ (*eat-6*^rt251/+^), worms exhibit signs of bradykinesia, characterised by a decreased rate of rhythmic contractions of their neuromuscular feeding organ (pharynx) relative to control worms (Sorkaç et al., 2016). However, *eat-6*^rt251/+^ and *eat-6*^rt252/+^ mutants both showed a reduced rate of pharyngeal pumping when aged to 8 days, indicating that the phenotypic deficit has a progressive nature (Sorkaç et al., 2016). Rhythmic contractions of the pharyngeal muscle pump liquid and suspended particles (bacterial food) in through the mouth, concentrate the bacteria and expel the excess liquid back out of the mouth. Feeding behaviour is relevant to AHC because patients can have difficulties in feeding and swallowing, whereas missed meals can trigger paroxysmal episodes (Neville and Ninan, 2007). By comparison, knockdown of *Atp1a3* expression in hypothalamic neurones was found to reduce food intake following fasting in rats (Kurita et al., 2015).

In the presence of 1 mM aldicarb, an acetylcholinesterase inhibitor that leads to acetylcholine accumulation at the neuromuscular junction and paralysis of *C. elegans* over time, *eat-6*^rt251/+^ and *eat-6*^rt252/+^ worms show paralysis at a significantly faster rate than WT worms (Sorkaç et al., 2016). This hypersensitivity to aldicarb is indicative of defects in neuromuscular junction function. The ataxia-relevant phenotypes of the *eat-6* mutants modelling the ARA-related G316S mutation suggest that *C. elegans* may be a useful model organism for the *in vivo* study of other mutations causing *ATP1A3*-related disorders.

## Concluding remarks and future perspectives

As behavioural abnormalities are the core symptoms of *ATP1A3*-related disorders, studies including behavioural measures in intact living animals are a powerful way to help resolve the pathophysiology of *ATP1A3*-related disorders. Genetically altered NKA α_3_ mouse models have enhanced our understanding of the phenotypic effects of mutations causing *ATP1A3*-related disorders. The replication of AHC features in *Myshkin*, Mashlool, Matoub and *Atp1a3*^D801Y^ mice (Table 3), and the access provided to their neurophysiology, highlight these models as valuable tools for the exploration of pathophysiological mechanisms and potential treatments. Nonetheless, decades of research on other neurological disorders, such as Alzheimer's disease, have shown that therapeutics that are disease modifying in mice are not necessarily disease modifying in human patients (Breijyeh and Karaman, 2020), perhaps reflecting some intrinsic physiological differences between humans and mice.

By comparison, the non-mammalian model systems are disadvantaged by their greater genetic and physiological distance from humans, although this is mitigated by the high conservation of NKA α_3_ relative to other proteins (Table 2). The neurological phenotypes exhibited by *Drosophila*, *C. elegans* and zebrafish with interference of *ATP1A3* orthologues demonstrate their capacity as useful tools to quickly and inexpensively assess the physiological and behavioural consequences of putative causative mutations in *ATP1A3*-related disorders. Moreover, their small size and short generation interval make them more suitable than mice for high-throughput screens of drugs and genetic modifiers. As there are welfare concerns surrounding the use of *Atp1a3* mutant mice with severe phenotypes (e.g. fatal seizures), the alternative models offer potential replacements to study aspects of *ATP1A3*-related disorders, under the principles of replacement, refinement and reduction (3Rs) in animal research.

Although the translational potential of invertebrate models is less obvious compared with mice, *Drosophila* models, in particular, have demonstrated their utility in drug discovery programmes for fragile X syndrome (Monyak et al., 2017) and *KRAS*-mutant metastatic colorectal cancer (Bangi et al., 2019). However, a consequence of the advent of CRISPR/Cas9 gene editing (Komor et al., 2017) is that genetic alteration of NKA α_3_ is no longer restricted to conventional ‘genetically tractable’ model organisms. There is now scope to generate NKA α_3_-mutated models from a wider range of animal species, including larger mammals, such as rabbits (Sui et al., 2018), and non-human primates, such as marmosets (Kumita et al., 2019). Given the current lack of published animal models for CAPOS, RECA and other *ATP1A3*-related disorders (Table 1), and the paucity of effective therapies, it is likely that NKA α_3_ animal models will continue to play an important role in *ATP1A3*-related disorder research in the years ahead.

## Supplementary Material

Supplementary information

## References

[DMM048938C1] Albers, R. W. (1967). Biochemical aspects of active transport. *Annu. Rev. Biochem.* 36, 727-756. 10.1146/annurev.bi.36.070167.003455 18257736

[DMM048938C2] Anselm, I. A., Sweadner, K. J., Gollamudi, S., Ozelius, L. J. and Darras, B. T. (2009). Rapid-onset dystonia-parkinsonism in a child with a novel atp1a3 gene mutation. *Neurology* 73, 400-401. 10.1212/WNL.0b013e3181b04acd 19652145 PMC2833268

[DMM048938C3] Arystarkhova, E., Haq, I. U., Luebbert, T., Mochel, F., Saunders-Pullman, R., Bressman, S. B., Feschenko, P., Salazar, C., Cook, J. F., Demarest, S. et al. (2019). Factors in the disease severity of *ATP1A3* mutations: impairment, misfolding, and allele competition. *Neurobiol. Dis.* 132, 104577. 10.1016/j.nbd.2019.104577 31425744 PMC7397496

[DMM048938C4] Ashmore, L. J., Hrizo, S. L., Paul, S. M., Van Voorhies, W. A., Beitel, G. J. and Palladino, M. J. (2009). Novel mutations affecting the Na, K ATPase alpha model complex neurological diseases and implicate the sodium pump in increased longevity. *Hum. Genet.* 126, 431-447. 10.1007/s00439-009-0673-2 19455355 PMC2791699

[DMM048938C5] Azarias, G., Kruusmägi, M., Connor, S., Akkuratov, E. E., Liu, X. L., Lyons, D., Brismar, H., Broberger, C. and Aperia, A. (2013). A specific and essential role for Na,K-ATPase α3 in neurons co-expressing α1 and α3. *J. Biol. Chem.* 288, 2734-2743. 10.1074/jbc.M112.425785 23195960 PMC3554939

[DMM048938C6] Balestrini, S., Mikati, M. A., Álvarez-García-Rovés, R., Carboni, M., Hunanyan, A. S., Kherallah, B., McLean, M., Prange, L., De Grandis, E., Gagliardi, A. et al. (2020). Cardiac phenotype in *ATP1A3*-related syndromes: a multicenter cohort study. *Neurology* 95, e2866-e2879. 10.1212/WNL.0000000000010794 32913013 PMC7734736

[DMM048938C7] Bangi, E., Ang, C., Smibert, P., Uzilov, A. V., Teague, A. G., Antipin, Y., Chen, R., Hecht, C., Gruszczynski, N., Yon, W. J. et al. (2019). A personalized platform identifies trametinib plus zoledronate for a patient with KRAS-mutant metastatic colorectal cancer. *Sci. Adv.* 5, eaav6528. 10.1126/sciadv.aav6528 31131321 PMC6531007

[DMM048938C8] Blanco-Arias, P., Einholm, A. P., Mamsa, H., Concheiro, C., Gutiérrez-de-Terán, H., Romero, J., Toustrup-Jensen, M. S., Carracedo, A., Jen, J. C., Vilsen, B. et al. (2009). A C-terminal mutation of ATP1A3 underscores the crucial role of sodium affinity in the pathophysiology of rapid-onset dystonia-parkinsonism. *Hum. Mol. Genet.* 18, 2370-2377. 10.1093/hmg/ddp170 19351654

[DMM048938C9] Boelman, C., Lagman-Bartolome, A. M., MacGregor, D. L., McCabe, J., Logan, W. J. and Minassian, B. A. (2014). Identical ATP1A3 mutation causes alternating hemiplegia of childhood and rapid-onset dystonia parkinsonism phenotypes. *Pediatr. Neurol.* 51, 850-853. 10.1016/j.pediatrneurol.2014.08.015 25439493

[DMM048938C10] Bourgeois, M., Aicardi, J. and Goutières, F. (1993). Alternating hemiplegia of childhood. *J. Pediatr.* 122, 673-679. 10.1016/S0022-3476(06)80003-X 8496742

[DMM048938C11] Brand, A. H. and Perrimon, N. (1993). Targeted gene expression as a means of altering cell fates and generating dominant phenotypes. *Development* 118, 401-415. 10.1242/dev.118.2.401 8223268

[DMM048938C12] Brashear, A., Butler, I. J., Hyland, K., Farlow, M. R. and Dobyns, W. B. (1998). Cerebrospinal fluid homovanillic acid levels in rapid-onset dystonia-parkinsonism. *Ann. Neurol.* 43, 521-526. 10.1002/ana.410430417 9546335

[DMM048938C13] Brashear, A., Mulholland, G. K., Zheng, Q.-H., Farlow, M. R., Siemers, E. R. and Hutchins, G. D. (1999). PET imaging of the pre-synaptic dopamine uptake sites in rapid-onset dystonia-parkinsonism (RDP). *Mov. Disord.* 14, 132-137. 10.1002/1531-8257(199901)14:1<132::AID-MDS1022>3.0.CO;2-J 9918356

[DMM048938C14] Brashear, A., Dobyns, W. B., de Carvalho Aguiar, P., Borg, M., Frijns, C. J. M., Gollamudi, S., Green, A., Guimaraes, J., Haake, B. C., Klein, C. et al. (2007). The phenotypic spectrum of rapid-onset dystonia–parkinsonism (RDP) and mutations in the *ATP1A3* gene. *Brain* 130, 828-835. 10.1093/brain/awl340 17282997

[DMM048938C15] Brashear, A., Cook, J. F., Hill, D. F., Amponsah, A., Snively, B. M., Light, L., Boggs, N., Suerken, C. K., Stacy, M., Ozelius, L. et al. (2012a). Psychiatric disorders in rapid-onset dystonia-parkinsonism. *Neurology* 79, 1168-1173. 10.1212/WNL.0b013e3182698d6c 22933743 PMC3525305

[DMM048938C16] Brashear, A., Mink, J. W., Hill, D. F., Boggs, N., McCall, W. V., Stacy, M. A., Snively, B., Light, L. S., Sweadner, K. J., Ozelius, L. J. et al. (2012b). *ATP1A3* mutations in infants: a new rapid-onset dystonia-Parkinsonism phenotype characterized by motor delay and ataxia. *Dev. Med. Child Neurol.* 54, 1065-1067. 10.1111/j.1469-8749.2012.04421.x 22924536 PMC3465467

[DMM048938C17] Breijyeh, Z. and Karaman, R. (2020). Comprehensive review on Alzheimer's disease: causes and treatment. *Molecules* 25, 5789. 10.3390/molecules25245789 PMC7764106 33302541

[DMM048938C18] Brown, G. R., Hem, V., Katz, K. S., Ovetsky, M., Wallin, C., Ermolaeva, O., Tolstoy, I., Tatusova, T., Pruitt, K. D., Maglott, D. R. et al. (2015). Gene: a gene-centered information resource at NCBI. *Nucleic Acids Res.* 43, D36-D42. 10.1093/nar/gku1055 25355515 PMC4383897

[DMM048938C19] Chang, I. J., Adam, M. P., Jayadev, S., Bird, T. D., Natarajan, N. and Glass, I. A. (2018). Novel pregnancy-triggered episodes of CAPOS syndrome. *Am. J. Med. Genet. A* 176, 235-240. 10.1002/ajmg.a.38502 29090527 PMC5726903

[DMM048938C20] Chaumette, B., Ferrafiat, V., Ambalavanan, A., Goldenberg, A., Dionne-Laporte, A., Spiegelman, D., Dion, P. A., Gerardin, P., Laurent, C., Cohen, D. et al. (2020). Missense variants in *ATP1A3* and *FXYD* gene family are associated with childhood-onset schizophrenia. *Mol. Psychiatry* 25, 821-830. 10.1038/s41380-018-0103-8 29895895 PMC6291354

[DMM048938C21] Clapcote, S. J., Duffy, S., Xie, G., Kirshenbaum, G., Bechard, A. R., Schack, V. R., Petersen, J., Sinai, L., Saab, B. J., Lerch, J. P. et al. (2009). Mutation I810N in the α3 isoform of Na^+^,K^+^-ATPase causes impairments in the sodium pump and hyperexcitability in the CNS. *Proc. Natl. Acad. Sci. USA* 106, 14085-14090. 10.1073/pnas.0904817106 19666602 PMC2729024

[DMM048938C22] Cook, J. F., Hill, D. F., Snively, B. M., Boggs, N., Suerken, C. K., Haq, I., Stacy, M., McCall, W. V., Ozelius, L. J., Sweadner, K. J. et al. (2014). Cognitive impairment in rapid-onset dystonia-parkinsonism. *Mov. Disord.* 29, 344-350. 10.1002/mds.25790 24436111 PMC3960305

[DMM048938C23] Dard, R., Mignot, C., Durr, A., Lesca, G., Sanlaville, D., Roze, E. and Mochel, F. (2015). Relapsing encephalopathy with cerebellar ataxia related to an *ATP1A3* mutation. *Dev. Med. Child Neurol.* 57, 1183-1186. 10.1111/dmcn.12927 26400718

[DMM048938C24] DeAndrade, M. P., Yokoi, F., van Groen, T., Lingrel, J. B. and Li, Y. (2011). Characterization of *Atp1a3* mutant mice as a model of rapid-onset dystonia with parkinsonism. *Behav. Brain Res.* 216, 659-665. 10.1016/j.bbr.2010.09.009 20850480 PMC2981691

[DMM048938C25] de Carvalho Aguiar, P., Sweadner, K. J., Penniston, J. T., Zaremba, J., Liu, L., Caton, M., Linazasoro, G., Borg, M., Tijssen, M. A. J., Bressman, S. B. et al. (2004). Mutations in the Na^+^/K^+^-ATPase α3 gene *ATP1A3* are associated with rapid-onset dystonia parkinsonism. *Neuron* 43, 169-175. 10.1016/j.neuron.2004.06.028 15260953

[DMM048938C26] Del Bene, V. E. (1990). Temperature. In *Clinical Methods:The History, Physical, and Laboratory Examinations*, 3rd edn (ed. H. K. Walker, W. D. Hall and J. W. Hurst). Boston: Butterworths. 21250045

[DMM048938C27] Demos, M. K., van Karnebeek, C. D. M., Ross, C. J. D., Adam, S., Shen, Y., Zhan, S. H., Shyr, C., Horvath, G., Suri, M., Fryer, A. et al. (2014). A novel recurrent mutation in *ATP1A3* causes CAPOS syndrome. *Orphanet J. Rare Dis.* 9, 15. 10.1186/1750-1172-9-15 24468074 PMC3937150

[DMM048938C28] Dobyns, W. B., Ozelius, L. J., Kramer, P. L., Brashear, A., Farlow, M. R., Perry, T. R., Walsh, L. E., Kasarskis, E. J., Butler, I. J. and Breakefield, X. O. (1993). Rapid-onset dystonia-parkinsonism. *Neurology* 43, 2596-2602. 10.1212/WNL.43.12.2596 8255463

[DMM048938C29] Doğanlı, C., Beck, H. C., Ribera, A. B., Oxvig, C. and Lykke-Hartmann, K. (2013). α3Na^+^/K^+^-ATPase deficiency causes brain ventricle dilation and abrupt embryonic motility in zebrafish. *J. Biol. Chem.* 288, 8862-8874. 10.1074/jbc.M112.421529 23400780 PMC3610961

[DMM048938C30] Duat Rodriguez, A., Prochazkova, M., Santos Santos, S., Rubio Cabezas, O., Cantarin Extremera, V. and Gonzalez-Gutierrez-Solana, L. (2017). Early diagnosis of CAPOS syndrome before acute-onset ataxia—review of the literature and a new family. *Pediatr. Neurol.* 71, 60-64. 10.1016/j.pediatrneurol.2017.01.009 28483396

[DMM048938C31] Geering, K. (2001). The functional role of β subunits in oligomeric P-type ATPases. *J. Bioenerg. Biomembr.* 33, 425-438. 10.1023/A:1010623724749 11762918

[DMM048938C32] Geering, K., Beggah, A., Good, P., Girardet, S., Roy, S., Schaer, D. and Jaunin, P. (1996). Oligomerization and maturation of Na,K-ATPase: functional interaction of the cytoplasmic NH2 terminus of the beta subunit with the alpha subunit. *J. Cell Biol.* 133, 1193-1204. 10.1083/jcb.133.6.1193 8682858 PMC2120891

[DMM048938C34] Goelz, M. F., Mahler, J., Harry, J., Myers, P., Clark, J., Thigpen, J. E. and Forsythe, D. B. (1998). Neuropathologic findings associated with seizures in FVB mice. *Lab Anim Sci.* 48, 34-37. 9517887

[DMM048938C35] Gurrieri, F., Tiziano, F. D., Zampino, G. and Neri, G. (2016). Recognizable facial features in patients with alternating hemiplegia of childhood. *Am. J. Med. Genet. A* 170, 2698-2705. 10.1002/ajmg.a.37808 27312461

[DMM048938C36] Haglund, M. M. and Schwartzkroin, P. A. (1990). Role of Na-K pump potassium regulation and IPSPs in seizures and spreading depression in immature rabbit hippocampal slices. *J. Neurophysiol.* 63, 225-239. 10.1152/jn.1990.63.2.225 2313342

[DMM048938C37] Han, K. H., Oh, D. Y., Lee, S., Lee, C., Han, J. H., Kim, M. Y., Park, H. R., Park, M. K., Kim, N. K. D., Lee, J. et al. (2017). ATP1A3 mutations can cause progressive auditory neuropathy: a new gene of auditory synaptopathy. *Sci. Rep.* 7, 16504. 10.1038/s41598-017-16676-9 29184165 PMC5705773

[DMM048938C38] Haq, I. U., Snively, B. M., Sweadner, K. J., Suerken, C. K., Cook, J. F., Ozelius, L. J., Miller, C., McCall, W. V., Whitlow, C. T. and Brashear, A. (2019). Revising rapid-onset dystonia–parkinsonism: broadening indications for ATP1A3 testing. *Mov. Disord.* 34, 1528-1536. 10.1002/mds.27801 31361359 PMC6879786

[DMM048938C39] Harris, J. J., Jolivet, R. and Attwell, D. (2012). Synaptic energy use and supply. *Neuron* 75, 762-777. 10.1016/j.neuron.2012.08.019 22958818

[DMM048938C40] Heimer, G., Sadaka, Y., Israelian, L., Feiglin, A., Ruggieri, A., Marshall, C. R., Scherer, S. W., Ganelin-Cohen, E., Marek-Yagel, D., Tzadok, M. et al. (2015). CAOS—episodic cerebellar ataxia, areflexia, optic atrophy, and sensorineural hearing loss: a third allelic disorder of the *ATP1A3* gene. *J. Child Neurol.* 30, 1749-1756. 10.1177/0883073815579708 25895915

[DMM048938C41] Heinzen, E. L., Swoboda, K. J., Hitomi, Y., Gurrieri, F., Nicole, S., de Vries, B., Tiziano, F. D., Fontaine, B., Walley, N. M., Heavin, S. et al. (2012). *De novo* mutations in *ATP1A3* cause alternating hemiplegia of childhood. *Nat. Genet.* 44, 1030-1034. 10.1038/ng.2358 22842232 PMC3442240

[DMM048938C42] Helseth, A. R., Hunanyan, A. S., Adil, S., Linabarger, M., Sachdev, M., Abdelnour, E., Arehart, E., Szabo, M., Richardson, J., Wetsel, W. C. et al. (2018). Novel E815K knock-in mouse model of alternating hemiplegia of childhood. *Neurobiol. Dis.* 119, 100-112. 10.1016/j.nbd.2018.07.028 30071271

[DMM048938C43] Hoei-Hansen, C. E., Dali, C. Í., Lyngbye, T. J. B., Duno, M. and Uldall, P. (2014). Alternating hemiplegia of childhood in Denmark: clinical manifestations and *ATP1A3* mutation status. *Eur. J. Paediatr. Neurol.* 18, 50-54. 10.1016/j.ejpn.2013.08.007 24100174

[DMM048938C44] Holm, T. H., Isaksen, T. J., Glerup, S., Heuck, A., Bøttger, P., Füchtbauer, E.-M., Nedergaard, S., Nyengaard, J. R., Andreasen, M., Nissen, P. et al. (2016a). Cognitive deficits caused by a disease-mutation in the α3 Na^+^/K^+^-ATPase isoform. *Sci. Rep* 6, 31972. 10.1038/srep31972 27549929 PMC4994072

[DMM048938C45] Holm, R., Toustrup-Jensen, M. S., Einholm, A. P., Schack, V. R., Andersen, J. P. and Vilsen, B. (2016b). Neurological disease mutations of α3 Na^+^,K^+^-ATPase: Structural and functional perspectives and rescue of compromised function. *Biochim. Biophys. Acta (BBA) Bioenerg.* 1857, 1807-1828. 10.1016/j.bbabio.2016.08.009 27577505

[DMM048938C46] Hully, M., Ropars, J., Hubert, L., Boddaert, N., Rio, M., Bernardelli, M., Desguerre, I., Cormier-Daire, V., Munnich, A., de Lonlay, P. et al. (2017). Mosaicism in ATP1A3-related disorders: not just a theoretical risk. *Neurogenetics* 18, 23-28. 10.1007/s10048-016-0498-9 27726050

[DMM048938C47] Hunanyan, A. S., Fainberg, N. A., Linabarger, M., Arehart, E., Leonard, A. S., Adil, S. M., Helseth, A. R., Swearingen, A. K., Forbes, S. L., Rodriguiz, R. M. et al. (2015). Knock-in mouse model of alternating hemiplegia of childhood: Behavioral and electrophysiologic characterization. *Epilepsia* 56, 82-93. 10.1111/epi.12878 25523819

[DMM048938C48] Hunanyan, A. S., Helseth, A. R., Abdelnour, E., Kherallah, B., Sachdev, M., Chung, L., Masoud, M., Richardson, J., Li, Q., Nadler, J. V. et al. (2018). Mechanisms of increased hippocampal excitability in the *Mashl^+/−^* mouse model of Na+/K+-ATPase dysfunction. *Epilepsia* 59, 1455-1468. 10.1111/epi.14441 29889309

[DMM048938C49] Hunanyan, A. S., Kantor, B., Puranam, R. S., Elliott, C., McCall, A., Dhindsa, J., Pagadala, P., Wallace, K., Poe, J., Gunduz, T. et al. (2021). Adeno-associated virus-mediated gene therapy in the Mashlool, *Atp1a3^Mashl/+^*, mouse model of alternating hemiplegia of childhood. *Hum. Gene Ther.* 32, 405-419. 10.1089/hum.2020.191 33577387 PMC8182483

[DMM048938C50] Hundal, H. S., Marette, A., Mitsumoto, Y., Ramlal, T., Blostein, R. and Klip, A. (1992). Insulin induces translocation of the alpha 2 and beta 1 subunits of the Na^+^/K^+^-ATPase from intracellular compartments to the plasma membrane in mammalian skeletal muscle. *J. Biol. Chem.* 267, 5040-5043. 10.1016/S0021-9258(18)42725-1 1312081

[DMM048938C51] Ikeda, K., Satake, S., Onaka, T., Sugimoto, H., Takeda, N., Imoto, K. and Kawakami, K. (2013). Enhanced inhibitory neurotransmission in the cerebellar cortex of *Atp1a3*-deficient heterozygous mice. *J. Physiol.* 591, 3433-3449. 10.1113/jphysiol.2012.247817 23652595 PMC3717237

[DMM048938C52] Ikeda, K., Tienda, A. A., Harrison, F. E. and Kawakami, K. (2021). Decreased content of ascorbic acid (vitamin C) in the brain of knockout mouse models of Na^+^,K^+^-ATPase-related neurologic disorders. *PLoS ONE* 16, e0246678. 10.1371/journal.pone.0246678 33544780 PMC7864419

[DMM048938C53] Isaksen, T. J., Kros, L., Vedovato, N., Holm, T. H., Vitenzon, A., Gadsby, D. C., Khodakhah, K. and Lykke-Hartmann, K. (2017). Hypothermia-induced dystonia and abnormal cerebellar activity in a mouse model with a single disease-mutation in the sodium-potassium pump. *PLoS Genet.* 13, e1006763. 10.1371/journal.pgen.1006763 28472154 PMC5436892

[DMM048938C54] Ishii, A., Saito, Y., Mitsui, J., Ishiura, H., Yoshimura, J., Arai, H., Yamashita, S., Kimura, S., Oguni, H., Morishita, S. et al. (2013). Identification of *ATP1A3* mutations by exome sequencing as the cause of alternating hemiplegia of childhood in japanese patients. *PLoS ONE* 8, e56120. 10.1371/journal.pone.0056120 23409136 PMC3568031

[DMM048938C55] Ito, T., Narugami, M., Egawa, K., Yamamoto, H., Asahina, N., Kohsaka, S., Ishii, A., Hirose, S. and Shiraishi, H. (2018). Long-term follow up of an adult with alternating hemiplegia of childhood and a p.Gly755Ser mutation in the *ATP1A3* gene. *Brain Dev.* 40, 226-228. 10.1016/j.braindev.2017.11.007 29269014

[DMM048938C56] Jaffer, F., Fawcett, K., Sims, D., Heger, A., Houlden, H., Hanna, M. G., Kingston, H. and Sisodiya, S. M. (2017). Familial childhood-onset progressive cerebellar syndrome associated with the *ATP1A3* mutation. *Neurol Genet.* 3, e145. 10.1212/NXG.0000000000000145 28382329 PMC5367920

[DMM048938C57] Jasien, J. M., Bonner, M., D'alli, R., Prange, L., Mclean, M., Sachdev, M., Uchitel, J., Ricano, J., Smith, B. and Mikati, M. A. (2019). Cognitive, adaptive, and behavioral profiles and management of alternating hemiplegia of childhood. *Dev. Med. Child Neurol.* 61, 547-554. 10.1111/dmcn.14077 30362107

[DMM048938C58] Jones, D. H., Li, T. Y., Arystarkhova, E., Barr, K. J., Wetzel, R. K., Peng, J., Markham, K., Sweadner, K. J., Fong, G.-H. and Kidder, G. M. (2005). Na,K-ATPase from mice lacking the γ subunit (FXYD2) exhibits altered Na^+^ affinity and decreased thermal stability. *J. Biol. Chem.* 280, 19003-19011. 10.1074/jbc.M500697200 15755730

[DMM048938C59] Kamm, C., Fogel, W., Wächter, T., Schweitzer, K., Berg, D., Kruger, R., Freudenstein, D. and Gasser, T. (2008). Novel *ATP1A3* mutation in a sporadic RDP patient with minimal benefit from deep brain stimulation. *Neurology* 70, 1501-1503. 10.1212/01.wnl.0000310431.41036.e0 18413579

[DMM048938C60] Kamphuis, D. J., Koelman, H., Lees, A. J. and Tijssen, M. A. J. (2006). Sporadic rapid-onset dystonia–parkinsonism presenting as Parkinson's disease. *Mov. Disord.* 21, 118-119. 10.1002/mds.20695 16161139

[DMM048938C61] Kanai, R., Ogawa, H., Vilsen, B., Cornelius, F. and Toyoshima, C. (2013). Crystal structure of a Na^+^-bound Na^+^,K^+^-ATPase preceding the E1P state. *Nature* 502, 201-206. 10.1038/nature12578 24089211

[DMM048938C62] Kansagra, S., Ghusayni, R., Kherallah, B., Gunduz, T., McLean, M., Prange, L., Kravitz, R. M. and Mikati, M. A. (2019). Polysomnography findings and sleep disorders in children with alternating hemiplegia of childhood. *J. Clin. Sleep Med.* 15, 65-70. 10.5664/jcsm.7572 30621840 PMC6329557

[DMM048938C63] Kaplan, J. H. (2002). Biochemistry of Na,K-ATPase. *Annu. Rev. Biochem.* 71, 511-535. 10.1146/annurev.biochem.71.102201.141218 12045105

[DMM048938C64] Kirshenbaum, G. S., Clapcote, S. J., Duffy, S., Burgess, C. R., Petersen, J., Jarowek, K. J., Yücel, Y. H., Cortez, M. A., Snead, O. C., Vilsen, B. et al. (2011a). Mania-like behavior induced by genetic dysfunction of the neuron-specific Na^+^,K^+^-ATPase α3 sodium pump. *Proc. Natl. Acad. Sci. USA* 108, 18144-18149. 10.1073/pnas.1108416108 22025725 PMC3207708

[DMM048938C65] Kirshenbaum, G. S., Saltzman, K., Rose, B., Petersen, J., Vilsen, B. and Roder, J. C. (2011b). Decreased neuronal Na^+^,K^+^-ATPase activity in *Atp1a3* heterozygous mice increases susceptibility to depression-like endophenotypes by chronic variable stress. *Genes Brain Behav.* 10, 542-550. 10.1111/j.1601-183X.2011.00691.x 21418141

[DMM048938C66] Kirshenbaum, G. S., Dawson, N., Mullins, J. G. L., Johnston, T. H., Drinkhill, M. J., Edwards, I. J., Fox, S. H., Pratt, J. A., Brotchie, J. M., Roder, J. C. et al. (2013). Alternating hemiplegia of childhood-related neural and behavioural phenotypes in Na^+^,K^+^-ATPase α3 missense mutant mice. *PLoS ONE* 8, e60141. 10.1371/journal.pone.0060141 23527305 PMC3603922

[DMM048938C67] Kirshenbaum, G. S., Dachtler, J., Roder, J. C. and Clapcote, S. J. (2015). Characterization of cognitive deficits in mice with an alternating hemiplegia-linked mutation. *Behav. Neurosci.* 129, 822-831. 10.1037/bne0000097 26501181 PMC4655871

[DMM048938C68] Kirshenbaum, G. S., Dachtler, J., Roder, J. C. and Clapcote, S. J. (2016a). Transgenic rescue of phenotypic deficits in a mouse model of alternating hemiplegia of childhood. *Neurogenetics* 17, 57-63. 10.1007/s10048-015-0461-1 26463346 PMC4701769

[DMM048938C69] Kirshenbaum, G. S., Idris, N. F., Dachtler, J., Roder, J. C. and Clapcote, S. J. (2016b). Deficits in social behavioral tests in a mouse model of alternating hemiplegia of childhood. *J. Neurogenet.* 30, 42-49. 10.1080/01677063.2016.1182525 27276195 PMC4917910

[DMM048938C70] Komor, A. C., Badran, A. H. and Liu, D. R. (2017). CRISPR-based technologies for the manipulation of eukaryotic genomes. *Cell* 168, 20-36. 10.1016/j.cell.2016.10.044 27866654 PMC5235943

[DMM048938C71] Kumita, W., Sato, K., Suzuki, Y., Kurotaki, Y., Harada, T., Zhou, Y., Kishi, N., Sato, K., Aiba, A., Sakakibara, Y. et al. (2019). Efficient generation of Knock-in/Knock-out marmoset embryo via CRISPR/Cas9 gene editing. *Sci. Rep.* 9, 12719. 10.1038/s41598-019-49110-3 31481684 PMC6722079

[DMM048938C72] Kurita, H., Xu, K. Y., Maejima, Y., Nakata, M., Dezaki, K., Santoso, P., Yang, Y., Arai, T., Gantulga, D., Muroya, S. et al. (2015). Arcuate Na^+^,K^+^-ATPase senses systemic energy states and regulates feeding behavior through glucose-inhibited neurons. *Am. J. Physiol.-Endocrinol. Metab.* 309, E320-E333. 10.1152/ajpendo.00446.2014 26081283

[DMM048938C73] Kwong, A. K.-Y., Tsang, M. H.-Y., Fung, J. L.-F., Mak, C. C.-Y., Chan, K. L.-S., Rodenburg, R. J. T., Lek, M., Huang, S., Pajusalu, S., Yau, M.-M. et al. (2021). Exome sequencing in paediatric patients with movement disorders. *Orphanet J. Rare Dis.* 16, 32. 10.1186/s13023-021-01688-6 33446253 PMC7809769

[DMM048938C74] Lutsenko, S. and Kaplan, J. H. (1993). An essential role for the extracellular domain of the sodium-potassium-ATPase .beta.-subunit in cation occlusion. *Biochemistry* 32, 6737-6743. 10.1021/bi00077a029 8392370

[DMM048938C75] Maas, R. P., Schieving, J. H., Schouten, M., Kamsteeg, E.-J. and van de Warrenburg, B. P. C. (2016). The genetic homogeneity of CAPOS syndrome: four new patients with the c.2452G>A (p.Glu818Lys) mutation in the *ATP1A3* gene. *Pediatr. Neurol.* 59, 71-75.e1. 10.1016/j.pediatrneurol.2016.02.010 27091223

[DMM048938C76] Marzin, P., Mignot, C., Dorison, N., Dufour, L., Ville, D., Kaminska, A., Panagiotakaki, E., Dienpendaele, A.-S., Penniello, M.-J., Nougues, M.-C. et al. (2018). Early-onset encephalopathy with paroxysmal movement disorders and epileptic seizures without hemiplegic attacks: about three children with novel *ATP1A3* mutations. *Brain Dev.* 40, 768-774. 10.1016/j.braindev.2018.05.008 29861155

[DMM048938C77] McDonough, A. A., Geering, K. and Farley, R. A. (1990). The sodium pump needs its β subunit. *FASEB J.* 4, 1598-1605. 10.1096/fasebj.4.6.2156741 2156741

[DMM048938C78] McGrail, K. M., Phillips, J. M. and Sweadner, K. J. (1991). Immunofluorescent localization of three Na,K-ATPase isozymes in the rat central nervous system: both neurons and glia can express more than one Na,K-ATPase. *J. Neurosci.* 11, 381-391. 10.1523/JNEUROSCI.11-02-00381.1991 1846906 PMC6575210

[DMM048938C79] McLean, W. J., Smith, K. A., Glowatzki, E. and Pyott, S. J. (2009). Distribution of the Na,K-ATPase α subunit in the rat spiral ganglion and organ of corti. *J. Assoc. Res. Otolaryngol.* 10, 37-49. 10.1007/s10162-008-0152-9 19082858 PMC2644389

[DMM048938C80] Min, K.-T. and Benzer, S. (1997). Spongecake and eggroll: two hereditary diseases in *Drosophila* resemble patterns of human brain degeneration. *Curr. Biol.* 7, 885-888. 10.1016/S0960-9822(06)00378-2 9382801

[DMM048938C81] Mishra, N. K., Peleg, Y., Cirri, E., Belogus, T., Lifshitz, Y., Voelker, D. R., Apell, H.-J., Garty, H. and Karlish, S. J. D. (2011). FXYD proteins stabilize Na,K-ATPase. *J. Biol. Chem.* 286, 9699-9712. 10.1074/jbc.M110.184234 21228272 PMC3058982

[DMM048938C82] Miyatake, S., Kato, M., Kumamoto, T., Hirose, T., Koshimizu, E., Matsui, T., Takeuchi, H., Doi, H., Hamada, K., Nakashima, M. et al. (2021). De novo ATP1A3 variants cause polymicrogyria. *Sci. Adv.* 7, eabd2368. 10.1126/sciadv.abd2368 33762331 PMC7990330

[DMM048938C151] Monyak, R. E., Emerson, D., Schoenfeld, B. P., Zheng, X., Chambers, D. B., Rosenfelt, C., Langer, S., Hinchey, P., Choi, C. H., McDonald, T. V. et al. (2017). Insulin signaling misregulation underlies circadian and cognitive deficits in a Drosophila fragile X model. *Mol. Psychiatry* 22, 1140-1148. 10.1038/mp.2016.51 27090306 PMC5071102

[DMM048938C83] Morth, J. P., Pedersen, B. P., Toustrup-Jensen, M. S., Sørensen, T. L.-M., Petersen, J., Andersen, J. P., Vilsen, B. and Nissen, P. (2007). Crystal structure of the sodium–potassium pump. *Nature* 450, 1043-1049. 10.1038/nature06419 18075585

[DMM048938C84] Moseley, A. E., Williams, M. T., Schaefer, T. L., Bohanan, C. S., Neumann, J. C., Behbehani, M. M., Vorhees, C. V. and Lingrel, J. B. (2007). Deficiency in Na,K-ATPase α isoform genes alters spatial learning, motor activity, and anxiety in mice. *J. Neurosci* 27, 616-626. 10.1523/JNEUROSCI.4464-06.2007 17234593 PMC6672804

[DMM048938C85] Nakamura, Y., Hattori, A., Nakashima, M., Ieda, D., Hori, I., Negishi, Y., Ando, N., Matsumoto, N. and Saitoh, S. (2018). A *de novo* p.Arg756Cys mutation in *ATP1A3* causes a distinct phenotype with prolonged weakness and encephalopathy triggered by fever. *Brain Dev.* 40, 222-225. 10.1016/j.braindev.2017.09.010 29066118

[DMM048938C86] Neville, B. G. R. and Ninan, M. (2007). The treatment and management of alternating hemiplegia of childhood. *Dev. Med. Child Neurol.* 49, 777-780. 10.1111/j.1469-8749.2007.00777.x 17880649

[DMM048938C87] Nicita, F., Travaglini, L., Sabatini, S., Garavaglia, B., Panteghini, C., Valeriani, M., Bertini, E., Nardocci, N., Vigevano, F. and Capuano, A. (2016). Childhood-onset ATP1A3-related conditions: Report of two new cases of phenotypic spectrum. *Parkinsonism Relat. Disord.* 30, 81-82. 10.1016/j.parkreldis.2016.05.029 27268479

[DMM048938C88] Oblak, A. L., Hagen, M. C., Sweadner, K. J., Haq, I., Whitlow, C. T., Maldjian, J. A., Epperson, F., Cook, J. F., Stacy, M., Murrell, J. R. et al. (2014). Rapid-onset dystonia-parkinsonism associated with the I758S mutation of the *ATP1A3* gene: a neuropathologic and neuroanatomical study of four siblings. *Acta Neuropathol.* 128, 81-98. 10.1007/s00401-014-1279-x 24803225 PMC4059967

[DMM048938C89] Paciorkowski, A. R., McDaniel, S. S., Jansen, L. A., Tully, H., Tuttle, E., Ghoneim, D. H., Tupal, S., Gunter, S. A., Vasta, V., Zhang, Q. et al. (2015). Novel mutations in *ATP1A3* associated with catastrophic early life epilepsy, episodic prolonged apnea, and postnatal microcephaly. *Epilepsia* 56, 422-430. 10.1111/epi.12914 25656163 PMC4363281

[DMM048938C90] Palladino, M. J., Bower, J. E., Kreber, R. and Ganetzky, B. (2003). Neural dysfunction and neurodegeneration in *Drosophila* Na^+^/K^+^ ATPase alpha subunit mutants. *J. Neurosci.* 23, 1276-1286. 10.1523/JNEUROSCI.23-04-01276.2003 12598616 PMC6742270

[DMM048938C91] Panagiotakaki, E., Gobbi, G., Neville, B., Ebinger, F., Campistol, J., Nevšímalová, S., Laan, L., Casaer, P., Spiel, G., Giannotta, M. et al. (2010). Evidence of a non-progressive course of alternating hemiplegia of childhood: study of a large cohort of children and adults. *Brain* 133, 3598-3610. 10.1093/brain/awq295 20974617

[DMM048938C92] Panagiotakaki, E., De Grandis, E., Stagnaro, M., Heinzen, E. L., Fons, C., Sisodiya, S., de Vries, B., Goubau, C., Weckhuysen, S., Kemlink, D. et al. (2015). Clinical profile of patients with *ATP1A3* mutations in alternating hemiplegia of childhood—a study of 155 patients. *Orphanet J. Rare Dis.* 10, 123. 10.1186/s13023-015-0335-5 26410222 PMC4583741

[DMM048938C93] Paul, S. M., Palladino, M. J. and Beitel, G. J. (2007). A pump-independent function of the Na,K-ATPase is required for epithelial junction function and tracheal tube-size control. *Development* 134, 147-155. 10.1242/dev.02710 17164420 PMC1955469

[DMM048938C94] Post, R. L., Hegyvary, C. and Kume, S. (1972). Activation by adenosine triphosphate in the phosphorylation kinetics of sodium and potassium ion transport adenosine triphosphatase. *J. Biol. Chem.* 247, 6530-6540. 10.1016/S0021-9258(19)44725-X 4263199

[DMM048938C95] Potic, A., Nmezi, B. and Padiath, Q. S. (2015). CAPOS syndrome and hemiplegic migraine in a novel pedigree with the specific *ATP1A3* mutation. *J. Neurol. Sci.* 358, 453-456. 10.1016/j.jns.2015.10.002 26453127

[DMM048938C96] Prange, L., Pratt, M., Herman, K., Schiffmann, R., Mueller, D. M., McLean, M., Mendez, M. M., Walley, N., Heinzen, E. L., Goldstein, D. et al. (2020). D-DEMØ, a distinct phenotype caused by *ATP1A3* mutations. *Neurol. Genet.* 6, e466. 10.1212/NXG.0000000000000466 32802951 PMC7413631

[DMM048938C97] Rajarao, S. J. R., Canfield, V. A., Mohideen, M.-A. P. K., Yan, Y.-L., Postlethwait, J. H., Cheng, K. C. and Levenson, R. (2001). The repertoire of Na,K-ATPase alpha and beta subunit genes expressed in the zebrafish, *Danio rerio*. *Genome Res.* 11, 1211-1220. 10.1101/gr.186001 11435403 PMC311090

[DMM048938C98] Roenn, C. P., Li, M., Schack, V. R., Forster, I. C., Holm, R., Toustrup-Jensen, M. S., Andersen, J. P., Petrou, S. and Vilsen, B. (2019). Functional consequences of the CAPOS mutation E818K of Na^+^,K^+^-ATPase. *J. Biol. Chem.* 294, 269-280. 10.1074/jbc.RA118.004591 30409907 PMC6322875

[DMM048938C99] Rosewich, H., Thiele, H., Ohlenbusch, A., Maschke, U., Altmüller, J., Frommolt, P., Zirn, B., Ebinger, F., Siemes, H., Nürnberg, P. et al. (2012). Heterozygous *de-novo* mutations in ATP1A3 in patients with alternating hemiplegia of childhood: a whole-exome sequencing gene-identification study. *Lancet Neurol.* 11, 764-773. 10.1016/S1474-4422(12)70182-5 22850527

[DMM048938C100] Rosewich, H., Baethmann, M., Ohlenbusch, A., Gärtner, J. and Brockmann, K. (2014a). A novel *ATP1A3* mutation with unique clinical presentation. *J. Neurol. Sci.* 341, 133-135. 10.1016/j.jns.2014.03.034 24713507

[DMM048938C101] Rosewich, H., Ohlenbusch, A., Huppke, P., Schlotawa, L., Baethmann, M., Carrilho, I., Fiori, S., Lourenço, C. M., Sawyer, S., Steinfeld, R. et al. (2014b). The expanding clinical and genetic spectrum of ATP1A3-related disorders. *Neurology* 82, 945-955. 10.1212/WNL.0000000000000212 24523486

[DMM048938C102] Rosewich, H., Weise, D., Ohlenbusch, A., Gärtner, J. and Brockmann, K. (2014c). Phenotypic overlap of alternating hemiplegia of childhood and CAPOS syndrome. *Neurology* 83, 861-863. 10.1212/WNL.0000000000000735 25056583

[DMM048938C103] Sabouraud, P., Riquet, A., Spitz, M.-A., Deiva, K., Nevsimalova, S., Mignot, C., Lesca, G., Bednarek, N., Doummar, D., Pietrement, C. et al. (2019). Relapsing encephalopathy with cerebellar ataxia are caused by variants involving p.Arg756 in *ATP1A3*. *Eur. J. Paediatr. Neurol.* 23, 448-455. 10.1016/j.ejpn.2019.02.004 30862413

[DMM048938C104] Saito, Y., Sakuragawa, N., Sasaki, M., Sugai, K. and Hashimoto, T. (1998). A case of alternating hemiplegia of childhood with cerebellar atrophy. *Pediatr. Neurol.* 19, 65-68. 10.1016/S0887-8994(98)00016-2 9682890

[DMM048938C105] Sasaki, M., Sakuragawa, N. and Osawa, M. (2001). Long-term effect of flunarizine on patients with alternating hemiplegia of childhood in Japan. *Brain Dev.* 23, 303-305. 10.1016/S0387-7604(01)00229-7 11504600

[DMM048938C106] Sasaki, M., Sakuma, H., Fukushima, A., Yamada, K.-i., Ohnishi, T. and Matsuda, H. (2009). Abnormal cerebral glucose metabolism in alternating hemiplegia of childhood. *Brain Dev.* 31, 20-26. 10.1016/j.braindev.2008.03.008 18492605

[DMM048938C107] Sasaki, M., Ishii, A., Saito, Y., Morisada, N., Iijima, K., Takada, S., Araki, A., Tanabe, Y., Arai, H., Yamashita, S. et al. (2014). Genotype–phenotype correlations in alternating hemiplegia of childhood. *Neurology* 82, 482-490. 10.1212/WNL.0000000000000102 24431296

[DMM048938C108] Sasaki, M., Ishii, A., Saito, Y. and Hirose, S. (2017). Progressive brain atrophy in alternating hemiplegia of childhood. *Mov. Disord. Clin. Pract.* 4, 406-411. 10.1002/mdc3.12451 30363489 PMC6174487

[DMM048938C109] Schirinzi, T., Graziola, F., Cusmai, R., Fusco, L., Nicita, F., Elia, M., Travaglini, L., Bertini, E., Curatolo, P., Vigevano, F. et al. (2018a). *ATP1A3*-related epileptic encephalopathy responding to ketogenic diet. *Brain Dev.* 40, 433-438. 10.1016/j.braindev.2018.01.002 29395663

[DMM048938C110] Schirinzi, T., Graziola, F., Nicita, F., Travaglini, L., Stregapede, F., Valeriani, M., Curatolo, P., Bertini, E., Vigevano, F. and Capuano, A. (2018b). Childhood rapid-onset ataxia: expanding the phenotypic spectrum of *ATP1A3* mutations. *Cerebellum* 17, 489-493. 10.1007/s12311-018-0920-y 29397530

[DMM048938C111] Severino, M., Pisciotta, L., Tortora, D., Toselli, B., Stagnaro, M., Cordani, R., Morana, G., Zicca, A., Kotzeva, S., Zanaboni, C. et al. (2020). White matter and cerebellar involvement in alternating hemiplegia of childhood. *J. Neurol.* 267, 1300-1311. 10.1007/s00415-020-09698-3 31950366

[DMM048938C112] Shamraj, O. I. and Lingrel, J. B. (1994). A putative fourth Na^+^,K(+)-ATPase alpha-subunit gene is expressed in testis. *Proc. Natl. Acad. Sci. USA* 91, 12952-12956. 10.1073/pnas.91.26.12952 7809153 PMC45558

[DMM048938C113] Simmons, C. Q., Thompson, C. H., Cawthon, B. E., Westlake, G., Swoboda, K. J., Kiskinis, E., Ess, K. C. and George, A. L. (2018). Direct evidence of impaired neuronal Na/K-ATPase pump function in alternating hemiplegia of childhood. *Neurobiol. Dis.* 115, 29-38. 10.1016/j.nbd.2018.03.009 29567111

[DMM048938C114] Sival, D. A., Vansenne, F., Van der Hout, A. H., Tijssen, M. A. J. and de Koning, T. J. (2018). Fever-induced paroxysmal weakness and encephalopathy (FIPWE)—part of a phenotypic continuum in patients with *ATP1A3* mutations? *Pediatr. Neurol.* 81, 57-58. 10.1016/j.pediatrneurol.2017.12.009 29477659

[DMM048938C115] Smedemark-Margulies, N., Brownstein, C. A., Vargas, S., Tembulkar, S. K., Towne, M. C., Shi, J., Gonzalez-Cuevas, E., Liu, K. X., Bilguvar, K., Kleiman, R. J. et al. (2016). A novel de novo mutation in *ATP1A3* and childhood-onset schizophrenia. *Mol. Case Stud.* 2, a001008. 10.1101/mcs.a001008 PMC5002930 27626066

[DMM048938C116] Smith, R. S., Florio, M., Akula, S. K., Neil, J. E., Wang, Y., Hill, R. S., Goldman, M., Mullally, C. D., Reed, N., Bello-Espinosa, L. et al. (2021). Early role for a Na^+^,K^+^-ATPase (ATP1A3) in brain development. *Proc. Natl. Acad. Sci. USA* 118, e2023333118. 10.1073/pnas.2023333118 34161264 PMC8237684

[DMM048938C117] Sorkaç, A., Alcantara, I. C. and Hart, A. C. (2016). *In vivo* modelling of *ATP1A3* G316S-induced ataxia in *C. elegans* using CRISPR/Cas9-mediated homologous recombination reveals dominant loss of function defects. *PLoS ONE* 11, e0167963. 10.1371/journal.pone.0167963 27936181 PMC5148073

[DMM048938C118] Stavropoulos, D. J., Merico, D., Jobling, R., Bowdin, S., Monfared, N., Thiruvahindrapuram, B., Nalpathamkalam, T., Pellecchia, G., Yuen, R. K. C., Szego, M. J. et al. (2016). Whole-genome sequencing expands diagnostic utility and improves clinical management in paediatric medicine. *NPJ Genomic Med.* 1, 15012. 10.1038/npjgenmed.2015.12 PMC5447450 28567303

[DMM048938C119] Stenshorne, I., Rasmussen, M., Salvanos, P., Tallaksen, C. M. E., Bindoff, L. A. and Koht, J. (2019). Fever-related ataxia: a case report of CAPOS syndrome. *Cerebellum Ataxias* 6, 2. 10.1186/s40673-019-0096-3 31410291 PMC6368810

[DMM048938C120] Sugimoto, H., Ikeda, K. and Kawakami, K. (2018). *Atp1a3*-deficient heterozygous mice show lower rank in the hierarchy and altered social behavior. *Genes Brain Behav.* 17, e12435. 10.1111/gbb.12435 29057568

[DMM048938C121] Sui, T., Lau, Y. S., Liu, D., Liu, T., Xu, L., Gao, Y., Lai, L., Li, Z. and Han, R. (2018). A novel rabbit model of Duchenne muscular dystrophy generated by CRISPR/Cas9. *Dis. Model Mech.* 11, dmm032201. 10.1242/dmm.032201 29871865 PMC6031364

[DMM048938C122] Sun, B., Xu, P., Wang, W. and Salvaterra, P. M. (2001). In vivo modification of Na^+^,K^+^-ATPase activity in *Drosophila*. *Comp. Biochem. Physiol. B Biochem. Mol. Biol.* 130, 521-536. 10.1016/S1096-4959(01)00470-5 11691629

[DMM048938C123] Svetel, M., Ozelius, L. J., Buckley, A., Lohmann, K., Brajković, L., Klein, C. and Kostić, V. S. (2010). Rapid-onset dystonia-parkinsonism: case report. *J. Neurol.* 257, 472-474. 10.1007/s00415-009-5385-y 19936820

[DMM048938C124] Sweadner, K. J., Toro, C., Whitlow, C. T., Snively, B. M., Cook, J. F., Ozelius, L. J., Markello, T. C. and Brashear, A. (2016). *ATP1A3* mutation in adult rapid-onset ataxia. *PLoS ONE* 11, e0151429. 10.1371/journal.pone.0151429 26990090 PMC4798776

[DMM048938C125] Sweadner, K. J., Arystarkhova, E., Penniston, J. T., Swoboda, K. J., Brashear, A. and Ozelius, L. J. (2019). Genotype-structure-phenotype relationships diverge in paralogs *ATP1A1, ATP1A2*, and *ATP1A3*. *Neurol. Genet.* 5, e303. 10.1212/NXG.0000000000000303 30842972 PMC6384024

[DMM048938C126] Sweney, M. T., Silver, K., Gerard-Blanluet, M., Pedespan, J.-M., Renault, F., Arzimanoglou, A., Schlesinger-Massart, M., Lewelt, A. J., Reyna, S. P. and Swoboda, K. J. (2009). Alternating hemiplegia of childhood: early characteristics and evolution of a neurodevelopmental syndrome. *Pediatrics* 123, e534-e541. 10.1542/peds.2008-2027 19254988

[DMM048938C127] Sweney, M. T., Newcomb, T. M. and Swoboda, K. J. (2015). The expanding spectrum of neurological phenotypes in children with ATP1A3 mutations, alternating hemiplegia of childhood, rapid-onset Dystonia-Parkinsonism, CAPOS and beyond. *Pediatr. Neurol.* 52, 56-64. 10.1016/j.pediatrneurol.2014.09.015 25447930 PMC4352574

[DMM048938C128] Takata, A., Miyake, N., Tsurusaki, Y., Fukai, R., Miyatake, S., Koshimizu, E., Kushima, I., Okada, T., Morikawa, M., Uno, Y. et al. (2018). Integrative analyses of *de novo* mutations provide deeper biological insights into autism spectrum disorder. *Cell Rep.* 22, 734-747. 10.1016/j.celrep.2017.12.074 29346770

[DMM048938C129] Talsma, A. D., Chaves, J. F., LaMonaca, A., Wieczorek, E. D. and Palladino, M. J. (2014). Genome-wide screen for modifiers of Na^+^/K^+^ATPase alleles identifies critical genetic loci. *Mol. Brain* 7, 89. 10.1186/s13041-014-0089-3 25476251 PMC4302446

[DMM048938C130] Termsarasab, P., Yang, A. C. and Frucht, S. J. (2015). Intermediate phenotypes of *ATP1A3* mutations: phenotype-genotype correlations. *Tremor Other Hyperkinet Mov.* 5, 336. 10.5334/tohm.255 PMC4578012 26417536

[DMM048938C131] Thiadens, A. A. H. J., Phan, T. M. L., Zekveld-Vroon, R. C., Leroy, B. P., van den Born, L. I., Hoyng, C. B., Klaver, C. C. W., Roosing, S., Pott, J.-W. R., van Schooneveld, M. J. et al. (2012). Clinical course, genetic etiology, and visual outcome in cone and cone–rod dystrophy. *Ophthalmology* 119, 819-826. 10.1016/j.ophtha.2011.10.011 22264887

[DMM048938C132] Timothy, J. W. S., Klas, N., Sanghani, H. R., Al-Mansouri, T., Hughes, A. T. L., Kirshenbaum, G. S., Brienza, V., Belle, M. D. C., Ralph, M. R., Clapcote, S. J. et al. (2018). Circadian disruptions in the *Myshkin* mouse model of mania are independent of deficits in suprachiasmatic molecular clock function. *Biol. Psychiatry* 84, 827-837. 10.1016/j.biopsych.2017.04.018 28689605 PMC6218650

[DMM048938C133] Torres, A., Brownstein, C. A., Tembulkar, S. K., Graber, K., Genetti, C., Kleiman, R. J., Sweadner, K. J., Mavros, C., Liu, K. X., Smedemark-Margulies, N. et al. (2018). De novo *ATP1A3* and compound heterozygous *NLRP3* mutations in a child with autism spectrum disorder, episodic fatigue and somnolence, and muckle-wells syndrome. *Mol. Genet. Metab. Rep.* 16, 23-29. 10.1016/j.ymgmr.2018.06.001 29922587 PMC6005789

[DMM048938C134] Toustrup-Jensen, M. S., Einholm, A. P., Schack, V. R., Nielsen, H. N., Holm, R., Sobrido, M.-J., Andersen, J. P., Clausen, T. and Vilsen, B. (2014). Relationship between intracellular Na^+^ concentration and reduced Na^+^ affinity in Na^+^,K^+^-ATPase mutants causing neurological disease. *J. Biol. Chem.* 289, 3186-3197. 10.1074/jbc.M113.543272 24356962 PMC3916523

[DMM048938C135] Tranebjærg, L., Strenzke, N., Lindholm, S., Rendtorff, N. D., Poulsen, H., Khandelia, H., Kopec, W., Lyngbye, T. J. B., Hamel, C., Delettre, C. et al. (2018). The CAPOS mutation in *ATP1A3* alters Na/K-ATPase function and results in auditory neuropathy which has implications for management. *Hum. Genet.* 137, 111-127. 10.1007/s00439-017-1862-z 29305691

[DMM048938C136] Uchitel, J., Helseth, A., Prange, L., McLean, M., Ghusayni, R., Sachdev, M., Hunanyan, A. and Mikati, M. A. (2019). The epileptology of alternating hemiplegia of childhood. *Neurology* 93, e1248-e1259. 10.1212/WNL.0000000000008159 31484714

[DMM048938C137] Uchitel, J., Abdelnour, E., Boggs, A., Prange, L., Pratt, M., Bonner, M., Jasien, J., Dawson, G., Abrahamsen, T. and Mikati, M. A. (2020). Social impairments in alternating hemiplegia of childhood. *Dev. Med. Child Neurol.* 62, 820-826. 10.1111/dmcn.14473 32031250

[DMM048938C138] Uchitel, J., Wallace, K., Tran, L., Abrahamsen, T., Hunanyan, A., Prange, L., Jasien, J., Caligiuri, L., Pratt, M., Rikard, B. et al. (2021). Alternating hemiplegia of childhood: evolution over time and mouse model corroboration. *Brain Commun.* 3, fcab128. 10.1093/braincomms/fcab128 34396101 PMC8361420

[DMM048938C139] Verret, S. and Steele, J. C. (1971). Alternating hemiplegia in childhood: a report of eight patients with complicated migraine beginning in infancy. *Pediatrics* 47, 675-680. 5089756

[DMM048938C140] Vetro, A., Nielsen, H. N., Holm, R., Hevner, R. F., Parrini, E., Powis, Z., Møller, R. S., Bellan, C., Simonati, A., Lesca, G. et al. (2021). *ATP1A2*- and *ATP1A3*-associated early profound epileptic encephalopathy and polymicrogyria. *Brain* 144, 1435-1450. 10.1093/brain/awab052 33880529

[DMM048938C141] Viollet, L., Glusman, G., Murphy, K. J., Newcomb, T. M., Reyna, S. P., Sweney, M., Nelson, B., Andermann, F., Andermann, E., Acsadi, G. et al. (2015). Alternating Hemiplegia of Childhood: retrospective genetic study and genotype-phenotype correlations in 187 subjects from the US AHCF registry. *PLoS ONE* 10, e0127045. 10.1371/journal.pone.0127045 25996915 PMC4440742

[DMM048938C142] Weigand, K. M., Messchaert, M., Swarts, H. G. P., Russel, F. G. M. and Koenderink, J. B. (2014). Alternating hemiplegia of childhood mutations have a differential effect on Na^+^,K^+^-ATPase activity and ouabain binding. *Biochim. Biophys. Acta BBA Mol. Basis Dis.* 1842, 1010-1016. 10.1016/j.bbadis.2014.03.002 24631656

[DMM048938C143] Wilcox, R., Brænne, I., Brüggemann, N., Winkler, S., Wiegers, K., Bertram, L., Anderson, T. and Lohmann, K. (2015). Genome sequencing identifies a novel mutation in *ATP1A3* in a family with dystonia in females only. *J. Neurol.* 262, 187-193. 10.1007/s00415-014-7547-9 25359261

[DMM048938C144] Woo, A. L., James, P. F. and Lingrel, J. B. (2000). Sperm motility is dependent on a unique isoform of the Na,K-ATPase. *J. Biol. Chem.* 275, 20693-20699. 10.1074/jbc.M002323200 10764792

[DMM048938C145] Yang, X., Gao, H., Zhang, J., Xu, X., Liu, X., Wu, X., Wei, L. and Zhang, Y. (2014). ATP1A3 mutations and genotype-phenotype correlation of alternating hemiplegia of childhood in Chinese patients. *PLoS ONE* 9, e97274. 10.1371/journal.pone.0097274 24842602 PMC4026576

[DMM048938C146] Yang, X., Zhang, Y., Yuan, D., Xu, X., Li, S., Wei, L., Wu, Y., Xiong, H., Liu, X., Bao, X. et al. (2015). [ATP1A3 gene mutations in patients with alternating hemiplegia of childhood]. Zhonghua Er Ke Za Zhi Chin. *J. Pediatr.* 53, 835-839. 26758322

[DMM048938C147] Yano, S. T., Silver, K., Young, R., DeBrosse, S. D., Ebel, R. S., Swoboda, K. J. and Acsadi, G. (2017). Fever-induced paroxysmal weakness and encephalopathy, a new phenotype of ATP1A3 mutation. *Pediatr. Neurol.* 73, 101-105. 10.1016/j.pediatrneurol.2017.04.022 28647130

[DMM048938C148] Zahler, R., Brines, M., Kashgarian, M., Benz, E. J. and Gilmore-Hebert, M. (1992). The cardiac conduction system in the rat expresses the alpha 2 and alpha 3 isoforms of the Na^+^,K(+)-ATPase. *Proc. Natl. Acad. Sci. USA* 89, 99-103. 10.1073/pnas.89.1.99 1309618 PMC48183

[DMM048938C149] Zanotti-Fregonara, P., Vidailhet, M., Kas, A., Ozelius, L. J., Clot, F., Hindié, E., Ravasi, L., Devaux, J.-Y. and Roze, E. (2008). [123I]-FP-CIT and [99mTc]-HMPAO single photon emission computed tomography in a new sporadic case of rapid-onset dystonia–parkinsonism. *J. Neurol. Sci.* 273, 148-151. 10.1016/j.jns.2008.06.033 18675996

[DMM048938C150] Zhou, G.-H., Ma, Y., Li, M.-L., Zhou, X.-Y., Mou, H. and Jin, Z.-B. (2020). ATP1A3 mutation as a candidate cause of autosomal dominant cone-rod dystrophy. *Hum. Genet.* 139, 1391-1401. 10.1007/s00439-020-02182-y 32440726

