# Supplementary material for: Genetically altered animal models for ATP1A3-related disorders
Source: Dis Model Mech. 2021 Oct 6;14(10):dmm048938. doi: 10.1242/dmm.048938 (PMC8503543; doi:10.1242/dmm.048938)
Supplement: Supplementary information [file dmm-14-048938-s1.pdf]

**Table S1. *ATP1A3* mutations found in *ATP1A3*-related disorders other than AHC**

| Amino acid change | NKA $\alpha_3$ domain | Residue conservation | CDS change | Primary diagnosis (other than AHC) | References |
|---|---|---|---|---|---|
| V129M | M2 | M, D, C | c.385G>A | COS | Smedemark-Margulies et al., 2016 |
| S137del* | M2 | M, D | c.410_412del-CCT* | RDP (without parkinsonism) | Wilcox et al., 2015 |
| Q140H | M2 | M, D, C | c.420G>T | D-DEMØ | Prange et al., 2020 |
| I274T | M3 | M, D, C | c.821T>C | RDP | de Carvalho Aguiar et al., 2004; Kamphuis et al., 2006 |
| E277K** | M3 | M, D, C | c.829G>A | RDP | de Carvalho Aguiar et al., 2004 |
| L292R | M3 | M, D, C | c.875T>G | PMG | Vetro et al., 2021 |
| G316S | M4 | M, D, C | c.946G>A | ARA | Sweadner et al., 2016 |
| G316V | M4 | M, D, C | c.947G>T | PMG | Vetro et al., 2021 |
| I318M | M4 | M, D, C | c.954C>G | Parkinsonism with ID | Kwong et al., 2021 |
| E324G | M4 | M, D, C | c.971A>G | D-DEMØ | Prange et al., 2020 |
| G325D | M4 | M, D, C | c.974G>A | D-DEMØ | Prange et al., 2020 |
| L327del | M4 | M, D, C | c.979_981delCTG | RDP | Kamm et al., 2008 |
| G358V | P | M, D, C | c.1073G>T | EIEE | Paciorkowski et al., 2015 |
| T360R | P | M, D, C | c.1079C>G | D-DEMØ | Prange et al., 2020 |
| S361P | P | M, D, C | c.1081T>C | PMG | Vetro et al., 2021 |

Disease Models & Mechanisms • Supplementary information

| Amino acid change | NKA $\alpha_3$ domain | Residue conservation | CDS change | Primary diagnosis (other than AHC) | References |
|---|---|---|---|---|---|
| I363N | P | M, D, C | c.1088T>A | EIEE | Paciorkowski et al., 2015 |
| T370N** | P | M, D, C | c.1109C>A | RDP | Rosewich et al., 2014b |
| W382R | N | M, D, C | c.1144T>C | RDP | Rosewich et al., 2014b |
| D583Y | N | M, D, C | c.1747G>T | RDP (infantile) | Nicita et al., 2016 |
| D591V | N | M, D, C | c.1772A>T | adCORD | Zhou et al., 2020 |
| C596Y | P | M, D, C | c.1787G>A | PMG | Miyatake et al., 2021 |
| D609Y | P | M, D, C | c.1825G>T | Hypotonia with dyspneic episodes | Marzin et al., 2018 |
| D609Y | P | M, D, C | c.1825G>T | PMG | Vetro et al., 2021 |
| T613M | P | M, D, C | c.1838C>T | RDP | de Carvalho Aguiar et al., 2004 |
| A681T | P | M, D, C | c.2041G>A | ASD | Torres et al., 2018 |
| S684F | P | M, D, C | c.2051C>T | RDP | Svetel et al., 2010 |
| G706R** | P | M, D, C | c.2116G>A | EE with strabismus and autistic features | Hully et al., 2017 |
| N744K | P | M, D, C | c. 2232C>A | AHC/RDP intermediate | Sival et al., 2018 |
| R756C | M5 | M, D, C | c.2266C>T | RECA | Dard et al., 2015; Jaffer et al., 2017; Nakamura et al., 2018; Nicita et al., 2016; Sabouraud et |

Disease Models & Mechanisms • Supplementary information

| Amino acid change | NKA $\alpha_3$ domain | Residue conservation | CDS change | Primary diagnosis (other than AHC) | References |
|---|---|---|---|---|---|
| | | | | | al., 2019; Sival et al., 2018 |
| R756C | M5 | M, D, C | c.2266C>T | ASD | Takata et al., 2018 |
| R756C | M5 | M, D, C | c.2266C>T | CRA | Schirinzi et al., 2018b |
| R756H** | M5 | M, D, C | c.2267G>A | RDP (infantile) | Brashear et al., 2012b |
| R756H** | M5 | M, D, C | c.2267G>A | RECA | Sabouraud et al., 2019; Yano et al., 2017 |
| R756L | M5 | M, D, C | c.2267G>T | RECA | Yano et al., 2017 |
| I758S | M5 | M, D, C | c.2273T>G | RDP | de Carvalho Aguiar et al., 2004 |
| K764del | M5 | M, D, C | c.2290_2292delAAG | PMG | Vetro et al., 2021 |
| P775R | M5 | M, D, C | c.2324C>G/ | PMG | Vetro et al., 2021 |
| F780L | M5 | M, D, C | c.2338T>C | RDP | de Carvalho Aguiar et al., 2004 |
| D801N** | M6 | M, D, C | c.2401G>A | PMG | Vetro et al., 2021 |
| D801Y** | M6 | M, D, C | c.2401G>T | RDP | de Carvalho Aguiar et al., 2004 |
| A813V | M6 | M, D, C | c.2438T>C | COS | Chaumette et al., 2020 |
| E818K | L6–7 | M, D, C | c.2452G>A | CAPOS | Demos et al., 2014; Duat Rodriguez et al., 2017; Stavropoulos et al., 2016; |

Disease Models & Mechanisms • Supplementary information

| Amino acid change | NKA $\alpha_3$ domain | Residue conservation | CDS change | Primary diagnosis (other than AHC) | References |
|---|---|---|---|---|---|
| | | | | | Stenshorne et al., 2019 |
| E818K | L6–7 | M, D, C | c.2452G>A | CRA | Schirinzi et al., 2018b |
| Q851R | M7 | M, D, C | c.2552A>G | PMG | Smith et al., 2021 |
| G854_F856del | M7 | | c.2560_2568del | PMG | Miyatake et al., 2021 |
| F857del | M7 | M, D, C | c.2570_2572del | PMG | Miyatake et al., 2021; Vetro et al., 2021 |
| G867D | L7–8 | M, D, C | c.2600G>A | AHC/RDP intermediate | Rosewich et al., 2014a |
| D887Y | L7–8 | M, D | c.2659G>T | PMG (familial) | Vetro et al., 2021 |
| L888P | L7–8 | M, D | c.2663T>C | PMG with AHC | Panagiotakaki et al., 2015; Gurrieri et al., 2016; Vetro et al., 2021 |
| G893W | L7–8 | M, D, C | c.2677G>T | PMG | Vetro et al., 2021 |
| Q895P | L7–8 | M | c.2684A>C | PMG | Miyatake et al., 2021 |
| R901M | L7–8 | M, D, C | c.2702G>T | PMG | Smith et al., 2021 |
| D923N** | M8 | M, D, C | c.2767G>A | RDP | Zanotti-Fregonara et al., 2008 |
| D923N** | M8 | M, D, C | c.2767G>A | RDP (infantile) | Anselm et al., 2009; Brashear et al., 2012b |
| L924P | M8 | M, D, C | c.2771T>C | PMG | Smith et al., 2021 |

Disease Models & Mechanisms • Supplementary information

| Amino acid change | NKA $\alpha_3$ domain | Residue conservation | CDS change | Primary diagnosis (other than AHC) | References |
|---|---|---|---|---|---|
| L924P | M8 | M, D, C | c.2771T>C | EIEE | Arystarkhova et al., 2019 |
| E951K** | M9 | M, D, C | c.2851G>A | AHC/RDP intermediate | Termsarasab et al., 2015 |
| P972del | L9–10 | M, D | c.2915_2917delCTC | PMG | Vetro et al., 2021 |
| abolition splice donor site | | | c.2921+1G>A | PMG | Smith et al., 2021 |
| D992del | M10 | M, D, C | c.2976_2978del | PMG | Miyatake et al., 2021 |
| D992dup | M10 | M, D, C | c.2976_2978dupCGA | PMG | Vetro et al., 2021 |
| Y4_I994delinsFAHLHL | M10 | M, C | c.2972_2982delins | PMG | Miyatake et al., 2021 |
| I994_R995insHEI | M10 | M, C | c.2975_2983dup | PMG | Miyatake et al., 2021 |
| Y1013dup | C-term | M, D, C | c.3038_3040dup | RDP | Blanco-Arias et al., 2009 |

CDS, coding sequence; M, conserved in mouse *Atp1a3*; D, conserved in *D. melanogaster ATPα*; C, conserved in *C. elegans eat-6*; EE, epileptic encephalopathy; ID, intellectual disability. Nucleotide and residue numbering are based on *ATP1A3* variant 1/isoform 1 (NM_152296). *Reported as p.S148del and erroneously as c.443_445del-GAG with NKA $\alpha_3$ isoform 2 numbering in Wilcox et al. (2015). **Mutation also identified in AHC (Boelman et al., 2014; Heinzen et al., 2012; Hoei-Hansen et al., 2014; Panagiotakaki et al., 2015; Rosewich et al., 2014b; Viollet et al., 2015; Yang et al., 2014).

Disease Models & Mechanisms • Supplementary information

**Table S2. *ATP1A3* mutations found in AHC patients**

| Amino acid change | NKA $\alpha_3$ domain | Residue conservation | CDS change | No. of AHC cases† | % of AHC cases |
|---|---|---|---|---|---|
| S137F | M2 | M, D | c.410C>T | 3 | 0.61 |
| S137Y | M2 | M, D | c.410C>A | 6 | 1.22 |
| Q140L | M2 | M, D, C | c.419A>T | 2 | 0.41 |
| A264_A289delinsVLG* | A | M, D, C | c.791_866delinsTTCTGGG | 1 | 0.20 |
| I274N | M3 | M, D, C | c.821T>A | 7 | 1.42 |
| V322D | M4 | M, D, C | 965T>A | 1 | 0.20 |
| E324D** | M4 | M, D, C | c.972G>C | 1 | 0.20 |
| E324Q | M4 | M, D, C | c.970G>C | 1 | 0.20 |
| L326R | M4 | M, D, C | c.977T>G | 2 | 0.41 |
| abolition splice donor site | | | c.993+1_993+2del | 1 | 0.20 |
| C333F | M4 | M, D, C | c.998G>T | 4 | 0.81 |
| T335P | M4 | M, D, C | c.1003A>C | 1 | 0.20 |
| G358C | P | M, D, C | c.1072G>T | 1 | 0.20 |
| G358S | P | M, D, C | c.1072G>A | 1 | 0.20 |
| T370N | P | M, D, C | c.1109C>A | 1 | 0.20 |
| L371P | P | M, D, C | c.1112T>C | 1 | 0.20 |
| C596R | P | M, D, C | c.1786T>C | 2 | 0.41 |
| G706R | P | M, D, C | c.2116G>A | 1 | 0.20 |
| L715P | P | M, D, C | c.2144T>C | 1 | 0.20 |
| G755A | M5 | M, D, C | c. 2264G>C | 1 | 0.20 |
| G755C | M5 | M, D, C | c.2263G>T | 4 | 0.81 |
| G755S | M5 | M, D, C | c.2263G>A | 12 | 2.43 |
| G755V | M5 | M, D, C | c.2264G>T | 1 | 0.20 |
| R756H | M5 | M, D, C | c.2267G>A | 3 | 0.61 |
| L757P | M5 | M, D, C | c.2270T>C | 1 | 0.20 |
| N761H | M5 | M, D, C | c.2281A>C | 1 | 0.20 |
| Y768C | M5 | M, D, C | c.2303A>G | 1 | 0.20 |
| Y768H | M5 | M, D, C | c.2302T>C | 1 | 0.20 |
| T769P | M5 | M, D, C | c.2305A>C | 1 | 0.20 |
| L770R | M5 | M, D, C | c.2309T>G | 1 | 0.20 |
| T771I | M5 | M, D, C | c.2312C>T | 1 | 0.20 |
| T771N | M5 | M, D, C | c.2312C>A | 2 | 0.41 |
| S772R | M5 | M, D, C | c.2314A>C | 2 | 0.41 |
| S772R | M5 | M, D, C | c.2316C>A | 2 | 0.41 |
| S772R | M5 | M, D, C | c.2316C>G | 2 | 0.41 |
| N773I | M5 | M, D, C | c.2318A>T | 1 | 0.20 |
| N773S | M5 | M, D, C | c.2318A>G | 3 | 0.61 |
| N773T | M5 | M, D, C | c.2318A>C | 1 | 0.20 |
| D801E | M6 | M, D, C | c.2403T>A | 4 | 0.81 |
| **D801N** | **M6** | **M, D, C** | **c.2401G>A** | **194** | **39.35** |
| D801V | M6 | M, D, C | c.2402A>T | 1 | 0.20 |
| D801Y | M6 | M, D, C | c.2401G>T | 1 | 0.20 |
| L802P | M6 | M, D, C | c.2405T>C | 1 | 0.20 |
| T804I | M6 | M, D, C | c.2411C>T | 5 | 1.01 |

Disease Models & Mechanisms • Supplementary information

| Amino acid change | NKA $\alpha_3$ domain | Residue conservation | CDS change | No. of AHC cases[†] | % of AHC cases |
|---|---|---|---|---|---|
| D805E | M6 | M, D, C | c.2415C>G | 2 | 0.41 |
| D805H | M6 | M, D, C | c.2413G>C | 1 | 0.20 |
| D805N | M6 | M, D, C | c.2413G>A | 1 | 0.20 |
| M806K | M6 | M, D, C | c.2417T>A | 1 | 0.20 |
| M806R | M6 | M, D, C | c.2417T>G | 2 | 0.41 |
| P808L | M6 | M, D, C | c.2423C>T | 2 | 0.41 |
| I810F | M6 | M, D, C | c.2428A>T | 2 | 0.41 |
| I810N | M6 | M, D, C | c.2429T>A | 2 | 0.41 |
| I810S | M6 | M, D, C | c.2429T>G | 1 | 0.20 |
| S811P | M6 | M, D, C | c.2431T>C | 9 | 1.83 |
| **E815K** | **L6–7** | **M, D, C** | **c.2443G>A** | **107** | **21.70** |
| L839P | M7 | M, D, C | c.2516T>C | 3 | 0.61 |
| abolition splice donor site | | | c.2542+1:G>A | 4 | 0.81 |
| abolition splice donor site | | | c.2542+2T>C | 2 | 0.41 |
| R901T | L7–8 | M, D, C | c.2702G>C | 1 | 0.20 |
| V919del | M8 | M, D, C | c.2755_2757delGTC | 3 | 0.61 |
| V919del | M8 | M, D, C | c.2751_2753delTGT | 2 | 0.41 |
| D923N | M8 | M, D, C | c.2767G>A | 3 | 0.61 |
| D923Y | M8 | M, D, C | c.2767G>T | 2 | 0.41 |
| C927F | M8 | M, D | c.2780G>T | 3 | 0.61 |
| C927W | M8 | M, D | c.2781C>G | 1 | 0.20 |
| C927Y | M8 | M, D | c.2780G>A | 3 | 0.61 |
| **G947R** | **M9** | **M, D, C** | **c.2839G>A**<br>**c.2839G>C** | **44** | **8.92** |
| E951K | M9 | M, D, C | c.2851G>A | 2 | 0.41 |
| A955D | M9 | M, D, C | c.2864C>A | 2 | 0.41 |
| D992Y | M10 | M, D, C | c.2974G>T | 3 | 0.61 |
| | | | | *n*=493 | 100% |

CDS, coding sequence; M, transmembrane domain; P, phosphorylation domain; L, extracellular loop; M, conserved in mouse *Atp1a3*; D, conserved in *D. melanogaster ATPα*; C, conserved in *C. elegans eat-6*. Nucleotide and residue numbering are based on *ATP1A3* variant 1/isoform 1 (NM_152296). The three most common mutations (D801N, E815K and G974R) are shown in bold text. *p.A264_A289delinsVLG is an in-frame indel. **G324D was erroneously listed as Q324D by Viollet et al. (2015). ***Reported as c.2542-1:G>A in Heinzen et al. (2012). [†]Cases collated from cohorts of Heinzen et al. (2012), Rosewich et al. (2012), Ishii et al. (2013), Hoei-Hansen et al. (2014), Rosewich et al. (2014b), Sasaki et al. (2014), Yang et al. (2014), Panagiotakaki et al. (2015), Viollet et al. (2015).

Disease Models & Mechanisms • Supplementary information
